# Polystyrene nanoparticles promote endometrial cancer development through the ACSS2-mediated reprogramming of arachidonic acid metabolism

**DOI:** 10.1038/s41420-026-03071-5

**Published:** 2026-03-26

**Authors:** Xiangchun Huang, Li Xu, Jielin Wang, Jiachen Cheng, Jing Yuan, Xin Liu, Irina Larionova, Julia Kzhyshkowska, Shuo Chen, Yang Zhao

**Affiliations:** 1 Cancer Hospital of Dalian University of Technology, Cancer Hospital of China Medical University, Liaoning Cancer Hospital & Institute, Shenyang, China; 2 Department of Obstetrics and Gynecology, Department of Gynecologic Oncology Research Office, Guangzhou Key Laboratory of Targeted Therapy for Gynecologic Oncology, Guangdong Provincial Key Laboratory of Major Obstetric Diseases, Guangdong Provincial Clinical Research Center for Obstetrics and Gynecology, Guangdong-Hong Kong-Macao Greater Bay Area Higher Education Joint Laboratory of Maternal-Fetal Medicine, The Third Affiliated Hospital, Guangzhou Medical University, Guangzhou, China; 3 Department of Laboratory Medicine, The Second Affiliated Hospital of Guangdong Medical University, Zhanjiang, China; 4 Department of Breast Surgery, Cancer Hospital of Dalian University of Technology, Liaoning Cancer Hospital & Institute, Shenyang, China; 5 Laboratory of Translational Cellular and Molecular Biomedicine, National Research Tomsk State University, Tomsk, Russia; 6 Laboratory of Molecular Therapy of Cancer, Cancer Research Institute, Tomsk National Research Medical Center, Russian Academy of Sciences, Tomsk, Russia; 7 Institute of Transfusion Medicine and Immunology, Institute for Innate Immunoscience (MI3), Medical Faculty Mannheim, Heidelberg University, Mannheim, Germany

**Keywords:** Endometrial cancer, Mechanisms of disease

## Abstract

Microplastics and nanoplastics are commonly found in our everyday environments. So far, microplastics and nanoplastics have been detected in various tissues and bodily fluids, including hair, sputum, digestive tissue, lungs, blood, placental and endometrial tissue. Although some studies indicate that microplastics and nanoplastics can promote tumor development, their impact on endometrial cancer (EC) remains unclear. In this study, we examined the effect of polystyrene nanoplastics (PS-NPs) on EC development and explored the underlying pathogenic mechanisms. We observed the uptake and accumulation of PS-NPs in HEC-1B cells and EC organoids. Through cell and organoid experiments as well as mouse models, we demonstrated that PS-NP exposure can accelerate EC progression in vitro and in vivo. Next, through transcriptomic sequencing and targeted metabolomic sequencing, We found that adenosine 5’-monophosphate-activated protein kinase (AMPK) can activate ACSS2 and promote its nuclear translocation. The nuclear entry of ACSS2 is associated with increased levels of H3K9 acetylation, which may be a potential mechanism through which it regulates *PLA2G3* expression. *PLA2G3* mRNA levels are upregulated, increasing the production of arachidonic acid (AA), and ultimately leads to the epithelial-mesenchymal transition (EMT) in EC cells. The relevant molecular markers in this study can provide new strategies for early warning and targeted intervention, reducing the potential impact of PS-NPs on EC.

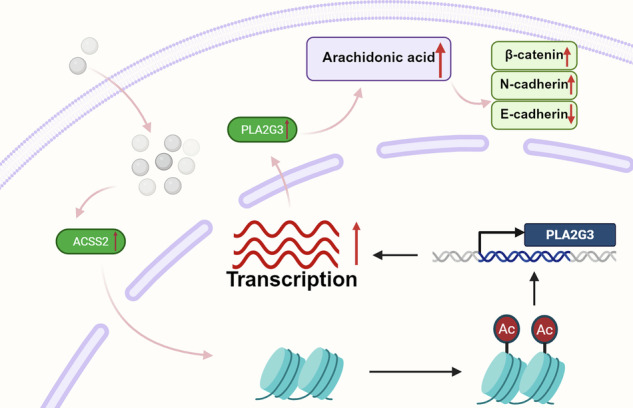

## Introduction

Due to the widespread use of plastics in our daily lives, we have now entered the “Age of Plastics” [1]. Plastic products have led to several technological advancements, enabling energy savings and providing many other benefits [2]. In 2023, the global production of plastics reached 400 million tons [3]. Environmental research has demonstrated that plastic debris undergoes fragmentation through multiple degradation pathways, including ultraviolet radiation, physical abrasion, and microbial activity, resulting in the generation of particulate plastic pollutants spanning size ranges from micrometer-scale (0.1 μm–5 mm, microplastics, MPs) to nanometer-scale (<0.1 μm, nanoplastics, NPs) that become dispersed throughout ecosystems [4, 5]. The growing presence of MPs and NPs in ecosystems and consumer products has generated substantial scientific and societal apprehension regarding their ecological and biomedical consequences. Humans are typically exposed to MPs through ingestion, inhalation, or dermal absorption [6–9]. The nanosized dimensions of NPs enhance their ability to penetrate physiological barriers and bioaccumulate in vital organs, posing greater toxicological risks compared to their MPs [10]. Nevertheless, experimental evidence demonstrates that MPs can also enter the human body, showing organ accumulation. So far, MPs and nano-microplastics have been detected in various tissues, in various cell types, and bodily fluids, such as hair, sputum, digestive tissues, lungs, blood, and placental tissues [11–19]. The accumulation of MPs and NPs in human cells can promote various pathological conditions, including cardiovascular events, asthma, liver inflammation, and cancer [20–24].

Endometrial cancer (EC) is the sixth most common form of cancer in women [25], accounting for 90% of uterine cancer cases [26]. Although deaths due to most solid tumors have declined over the past decade, the incidence of EC and related deaths in the United States has shown an increasing trend [27]. Several studies have revealed the link between MPs and malignant processes. For instance, Kim et al. found that exposure to MPs can lead to drug resistance in gastric cancer [28]. Meanwhile, Brynzak-Schreiber et al. discovered that MPs can be taken up by colorectal cancer cells, promoting their migration [29]. MP and NP can affect the female reproductive system through cellular and molecular pathways such as oxidative stress and inflammatory responses, and may accelerate carcinogenesis in the female reproductive system [30]. Qin et al. were the first to directly detect microplastics in human endometrial tissue [31]. However, the effects of MPs on EC have not been studied extensively, and our understanding of NPs toxicity in frontal EC remains very limited.

Metabolic reprogramming is a hallmark of cancer, and EC is associated with metabolic disorders [32–35]. In particular, the reprogramming of lipid metabolism can impact the development, invasiveness, metastasis, therapeutic resistance, and immune regulation of EC [36, 37]. Thus, targeting lipid metabolic pathways has emerged as a novel therapeutic strategy for EC.

Given these findings, in this study, we investigated whether polystyrene NPs (PS-NPs) promote the development of EC and explored the specific mechanisms involved in such pathogenic processes. Using human EC cell lines (HEC-1B and Ishikawa) as in vitro models, we systematically evaluated PS-NP-induced alterations in cellular proliferation and migratory behavior. And we used samples taken from clinical tissues to construct organoids in order to observe whether PS-NPs promote the growth of EC organoids. To validate our in vitro findings, we established a murine xenograft model to evaluate the pathophysiological effects of PS-NPs exposure. Tumor progression was quantitatively assessed through caliper measurements, while comprehensive histopathological examination of excised tumors was performed to characterize tissue-level alterations. The molecular basis of PS-NP effects was examined through combined transcriptomic and metabolomic approaches. This groundbreaking study establishes for the first time a comprehensive framework for understanding the pathological changes induced by PS-NPs in promoting EC progression, and it has important implications for precision medicine.

## Results

### Characterization and accumulation of PS-NPs in EC cells and organoids

**Fig. 1:**
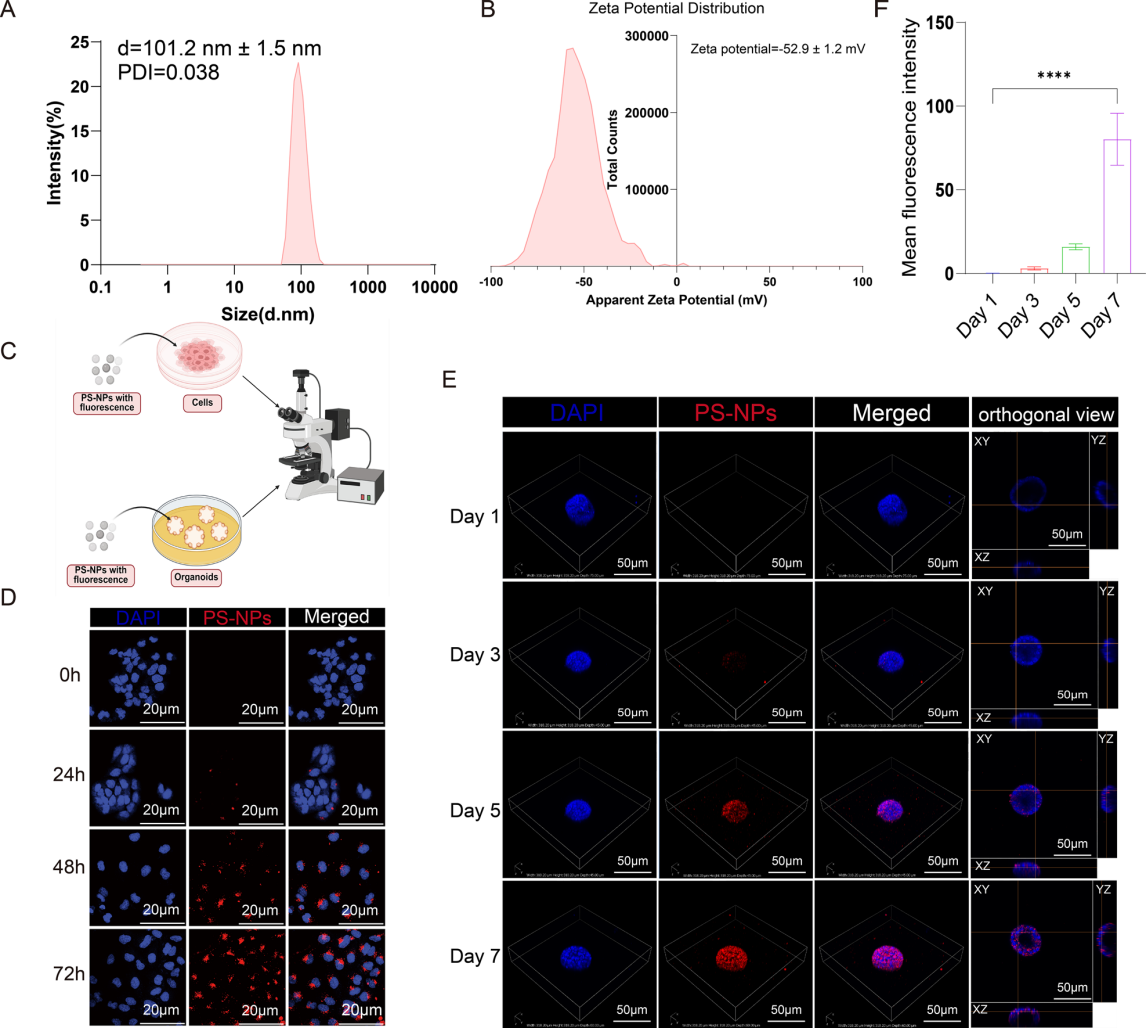
Characterization and Accumulation of PS-NPs in EC Cells and Organoids. The average hydrodynamic diameter of PS-NPs in DMEM medium was 101.2 nm ± 1.5 nm, with a PDI of 0.038 (**A**). The average zeta potential of PS-NPs in DMEM medium was -52.9 ± 1.2 mV (**B**). Co-culture of cells and organoids with fluorescent-bearing PS-NPs (**C**). Subcellular localization of PS-NPs in EC cells (**D**). 3D distribution of PS-NPs in EC organoids and cross-sectional view of PS-NPs with organoid structure (**E**). Quantitative analysis of the fluorescence intensity of PS-NPs within organoids over time (**F**). PS-NP fluorescence appears red and nuclei are stained blue by DAPI. Each experiment was performed with at least three biological replicates. For two-group comparisons, a t-test was applied. For comparisons across multiple groups, one-way or two-way ANOVA was employed. *p ≤ 0.05;**p ≤ 0.01; ***p ≤ 0.001; ****p ≤ 0.0001.

By dynamic light scattering (DLS) measurement, the average hydrodynamic diameter of PS-NPs in the cell culture medium used for experiments was 101.2 nm ± 1.5 nm, with a polydispersity index (PDI) of 0.038 (Fig. 1A), indicating that the particles were uniformly dispersed. DLS measurement also showed that the average zeta potential of PS-NPs in the experimental medium was −52.9 ± 1.2 mV (Fig. 1B), suggesting that the particle surfaces were negatively charged and that they were stable in solution. The endotoxin level in PS-NPs was measured by the gel-clot LAL assay and was determined to be <0.030 EU/mL (Supplementary Figure 1). To investigate whether PS-NPs can be taken up by EC cells and organoids, we monitored HEC-1B cells and organoids incubated with fluorescently labeled PS-NPs using microscope (Fig. 1C). After exposure to PS-NPs for 0, 24, 48, and 72 h, red fluorescent signals were detected around the DAPI-stained nuclei of HEC-1B cells, and their accumulation increased over time (Fig. 1D). Additionally, when EC organoids were exposed to PS-NPs, the red fluorescence signals within the organoids were found to gradually intensify over the 7-day incubation period (Fig. 1E, F).

### Long-term exposure to PS-NPs enhances the malignant biological behavior of EC cells and organoids

**Fig. 2:**
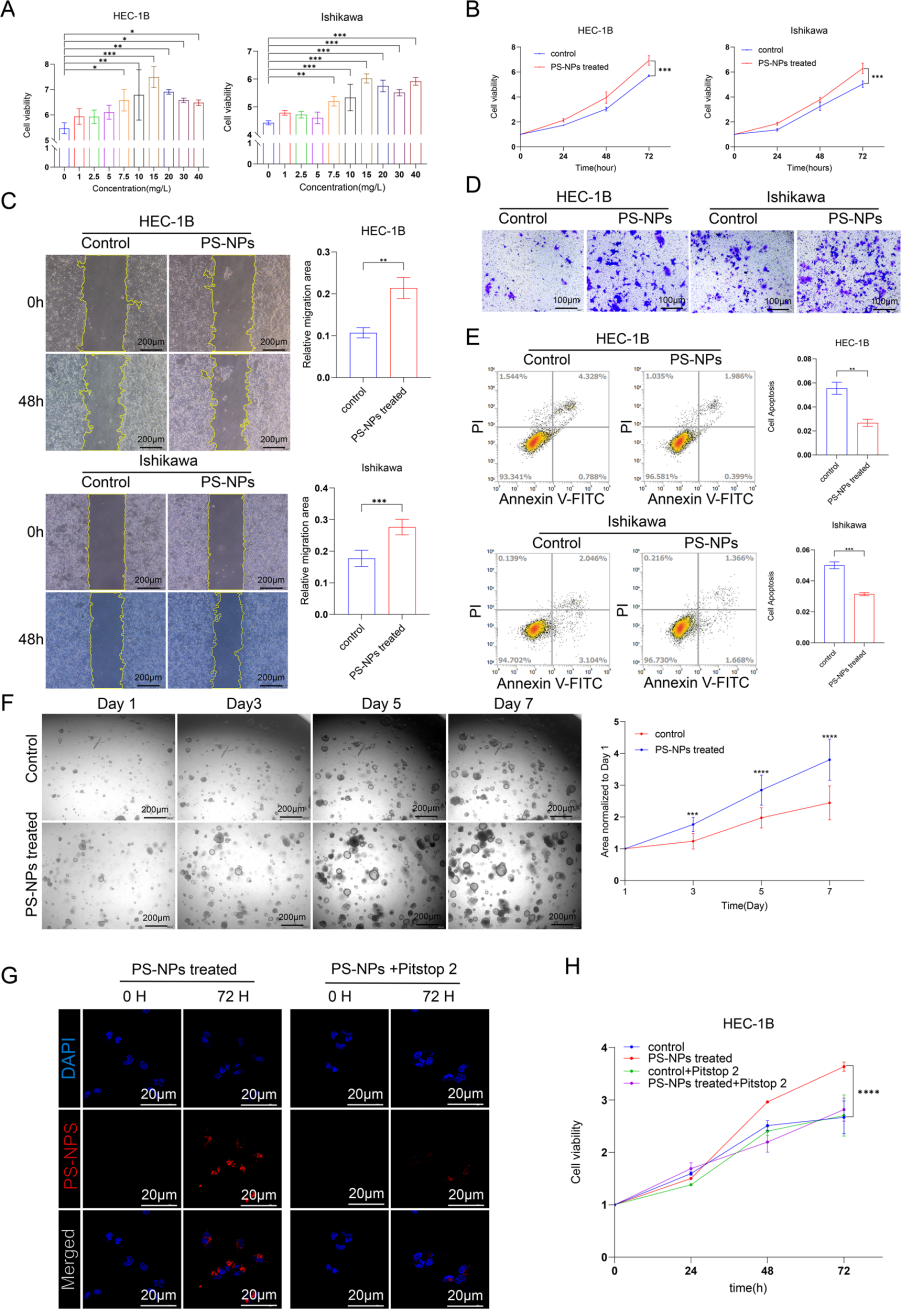
Long-term exposure to PS-NPs enhances the malignant biological behavior of EC cells and organoids. HEC-1B and Ishikawa proliferative capacity was enhanced after short time exposure to PS-NPs (**A**). Cell viability was increased after prolonged exposure to PS-NPs (**B**), cell migration (**C**) and invasion (**D**) were enhanced, and apoptosis was reduced (**E**). Compared with the control group, PS-NPs treatment for 7 days significantly accelerated organoid growth (**F**). The clathrin inhibitor Pitstop 2 significantly inhibited cell endocytosis of PS-NPs (**G**). Pitstop 2 treatment reversed the promoting effect of PS-NPs on EC cell growth (**H**). Each experiment was performed with at least three biological replicates. For two-group comparisons, a *t*-test was applied. For comparisons across multiple groups, one-way or two-way ANOVA was employed. Values are expressed as mean ± SD. *p ≤ 0.05;**p ≤ 0.01; ***p ≤ 0.001; ****p ≤ 0.0001.

Next, we examined the effects of PS-NPs on human EC cells. Wu et al. reported that FBS can influence the toxicity of PS-NPs [38]. Thus, we compared the viability of HEC-1B and Ishikawa cells treated with PS-NPs in FBS-free versus FBS-containing medium after 0, 24, 48, and 72 h. Notably, although the cell viability remained largely unchanged in the FBS-containing medium, it increased significantly in the FBS-free medium (Fig. 2A). Since cell culture in an FBS-containing medium better mimics in vivo conditions, we exposed the two EC cell lines to PS-NPs (15 mg/L) for 1 month to establish a long-term exposure model. We observed increased cell proliferation in both groups of cells exposed to PS-NPs over a prolonged period when compared to the primary cell lines (Fig. 2B). Additionally, long-term exposure to PS-NPs was found to enhance cell migration and invasion in both HEC-1B and Ishikawa cell lines (Fig. 2C, D). Notably, apoptosis rates were significantly lower in cells exposed to PS-NPs than in control cells (Fig. 2E). We treated EC organoids with PBS and PS-NPs separately for 7 consecutive days and measured their size. The results showed that the organoids treated with PS-NPs grew significantly faster than the control group (Fig. 2F). Next, we used the clathrin inhibitor Pitstop 2 and found that the uptake of nanoparticles by cells was significantly inhibited (Fig. 2G), and the promotion of EC growth by PS-NPs was also suppressed (Fig. 2H). PS-NPs promote the progression of EC after being endogenously taken up by EC cells and organoids.

### PS-NPs affect tumor growth in mice

**Fig. 3:**
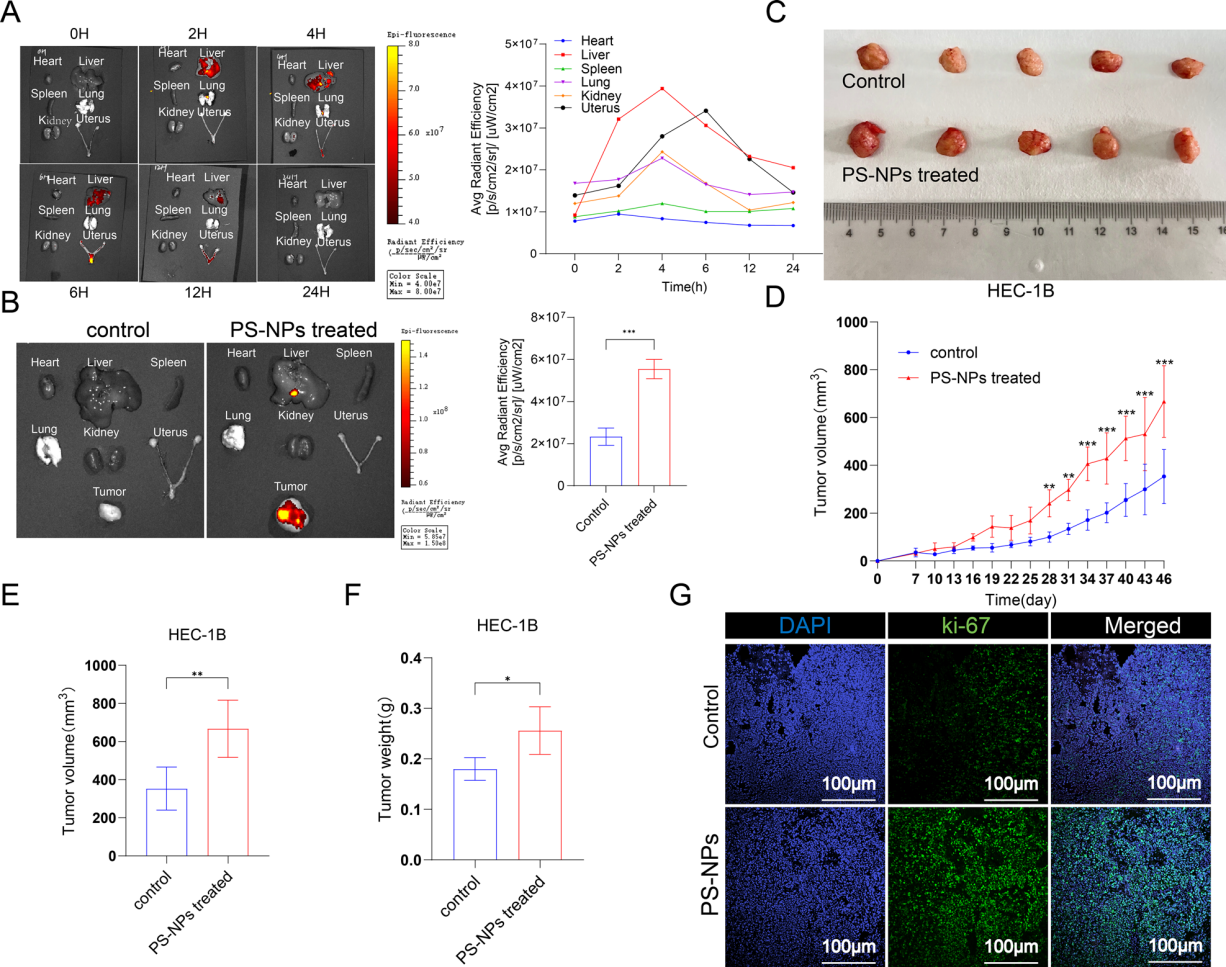
PS-NPs affect tumor growth in mice. Acute exposure: In vivo imaging after a single oral administration of fluorescent-labeled PS-NPs showed their distribution and accumulation in the liver, kidneys, and uterus (**A**). Chronic accumulation: After long-term daily gavage, significant accumulation of PS-NPs was observed in tumor tissues of tumor-bearing mice (**B**). Long-term exposure to PS-NPs (40 mg/L in drinking water) significantly promoted tumor volume increase in mice (**C**). After 46 days of exposure, the average tumor volume in the PS-NPs group (666.9 mm³) was approximately 1.89 times that of the control group (353.2 mm³) (**D**). Tumor volume and weight in the PS-NPs group were significantly higher than those in the control group (**E**, **F**). Immunofluorescence showed increased Ki-67 expression in the PS-NPs group (**G**). Each experiment was performed with at least three biological replicates. For two-group comparisons, a t-test was applied. For comparisons across multiple groups, one-way or two-way ANOVA was employed. Values are expressed as mean ± SD. *p ≤ 0.05;**p ≤ 0.01; ***p ≤ 0.001; ****p ≤ 0.0001.

To investigate the potential for dietary PS-NPs to enter systemic circulation and preferentially accumulate in the uterus and tumor tissues, we conducted acute distribution and long-term accumulation studies. Acute exposure was assessed by administering a single oral dose of fluorescently labeled PS-NPs and performing in vivo imaging at specified time points (0, 2, 4, 6, 12, and 24 h), which identified distribution and accumulation in the liver, kidneys, and uterus. We found that over time, PS-NPs accumulate in organs such as the liver, kidneys, lungs, and uterus. By 24 h, due to metabolic reasons, the fluorescence intensity in each organ almost returns to the level at 0 h (Fig. 3A). To evaluate chronic accumulation, tumor-bearing mice underwent prolonged daily gavage; in vivo imaging 24 h after cessation of dosing confirmed significant PS-NPs accumulation in tumor tissues (Fig. 3B). At the same time, we recorded changes in tumor volume in untreated mice and mice treated with PS-NPs over a prolonged period. Our findings showed that, compared to the control group, mice exposed to PS-NPs (40 mg/L) via drinking water exhibited significantly increased tumor growth (Fig. 3C). After 46 days of exposure to PS-NPs, the average tumor volume was 353.2 mm^3^ in the control group and 666.9 mm^3^ in the PS-NPs group (Fig. 3D). Moreover, tumor volume in the 40 mg/L PS-NP exposure group was 1.89 times that in the control group, and tumor weights were also heavier than control group (Fig. 3E, F). Notably, the immunofluorescence results showed that the PS-NPs-treated group had more expression of ki-67 than the control group (Fig. 3G). Moreover, there were no significant changes in the expression levels of estrogen receptor (ER) and progesterone receptor (PR) between the control group and the PS-NPs treatment group (Supplementary Figure 2A, B).

### PS-NPs promote the progression of EC by mediating the upregulation of acyl-CoA synthetase short-chain family member 2 (ACSS2)

**Fig. 4:**
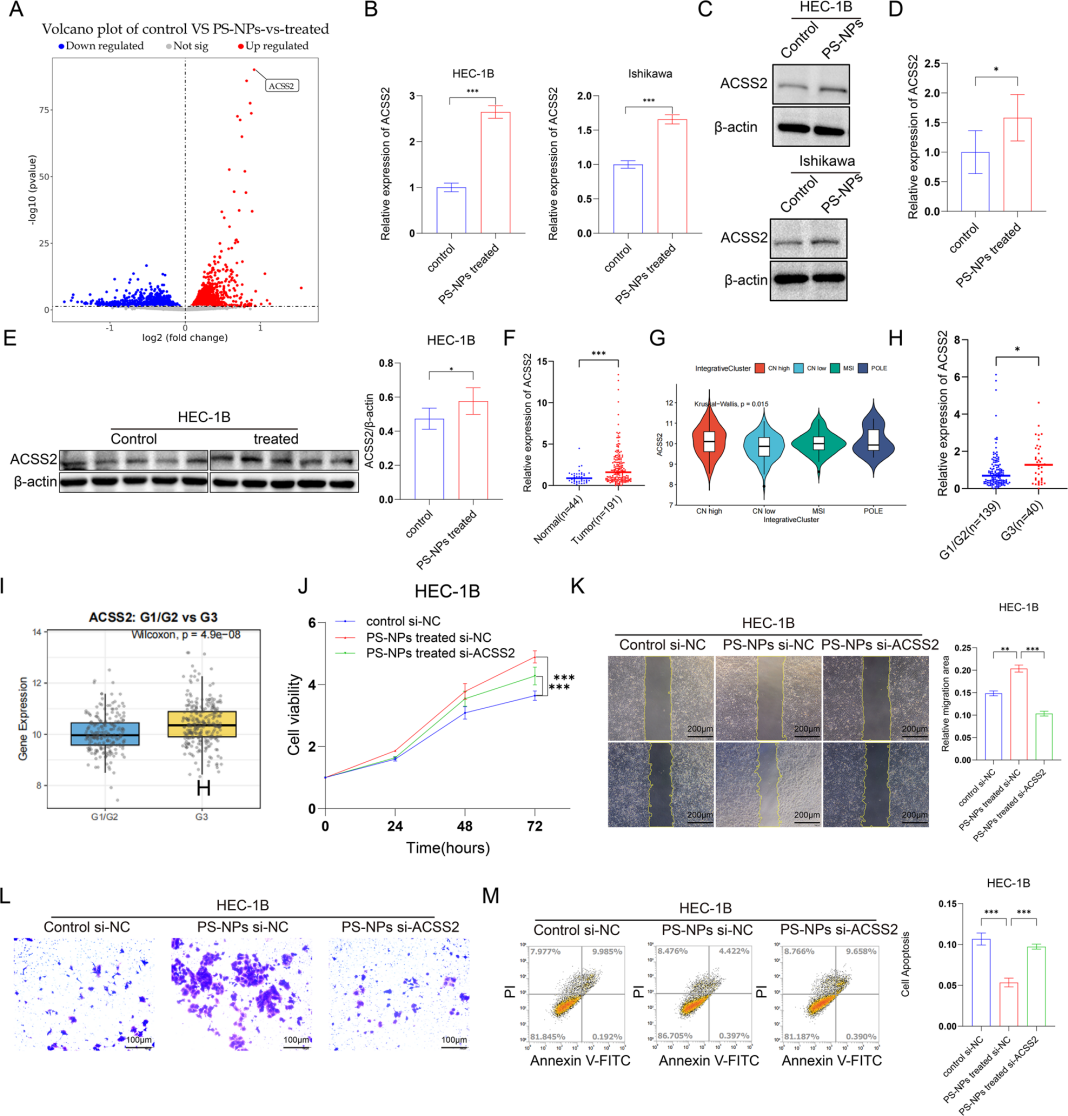
PS-NPs promote the progression of EC by mediating the upregulation of acyl-CoA synthetase short-chain family member 2 (ACSS2). Volcano plots showing differentially expressed genes with and without PS-NPs treatment (**A**). PS-NPs exposure upregulated ACSS2 mRNA and protein expression in HEC-1B and Ishikawa cells (**B**, **C**). In mice that ingested PS-NPs, the mRNA and protein levels of ACSS2 in tumor tissues were increased (**D**, **E**). q-PCR analysis showed that ACSS2 expression was significantly upregulated in EC tissues (*n* = 191) compared to normal tissues (*n* = 44) (*p* < 0.001) (**F**). TCGA data analysis indicated that high ACSS2 expression was associated with high copy number (CN high) subgroups (**G**). Analysis based on the TCGA database and our clinical cohort confirmed that ACSS2 expression is significantly higher in high-grade (G3) tumors compared to low-grade (G1/G2) tumors (**H**, **I**). In cell models with long-term PS-NPs exposure, knocking down ACSS2 reduced cell proliferation, migration, and invasion abilities, and promoted apoptosis (**J**–**M**). Each experiment was performed with at least three biological replicates. For two-group comparisons, a *t*-test was applied. For comparisons across multiple groups, one-way or two-way ANOVA was employed. Values are expressed as mean ± SD. *p ≤ 0.05; **p ≤ 0.01; ***p ≤ 0.001; ****p ≤ 0.0001.

EC is usually driven by hormones [39–42]. Thus we examined the estrogen- and progesterone-related genes *ESR1, PGR*, and *HOXA10* in cell lines exposed to PS-NPs as well as in tumors in vivo. We found that after treatment with PS-NPs, the mRNA levels of *ESR1, PGR, and HOXA10* in mouse tumors did not change. Compared with the control group, the mRNA and protein levels of ESR1, PGR, and HOXA10 in EC cell lines treated with PS-NPs were unchanged (Supplementary Figure 3A–J). In order to further explore the molecular mechanism through which PS-NPs affect the progression of EC, we subjected HEC-1B cells exposed to PS-NPs to RNA sequencing (RNA-seq). Then, we compared the transcriptomic profiles of these cells and untreated HEC-1B cells. The differentially expressed gene (DEG) analysis of HEC-1B cells after exposure to PS-NPs revealed the altered expression of 3,222 genes. These DEGs were identified based on adjusted P-values, and the gene *ACSS2*, which exhibited the lowest adjusted *P* value (*P* = 1.11 × 10^−86^), was prioritized for downstream functional analysis (Fig. 4A). Subsequently, validation experiments based on RT-qPCR and western blot analysis showed that *ACSS2* mRNA and ACSS2 protein levels were upregulated in both HEC-1B and Ishikawa cells after PS-NP exposure (Fig. 4B, C). Moreover, compared to control group, mice that had been fed with PS-NPs showed upregulation of *ACSS2* mRNA and ACSS2 protein levels (Fig. 4D, E). Analysis of our clinical cohort (44 normal endometrial and 191 endometrial carcinoma tissues) by qRT-PCR revealed that *ACSS2* mRNA expression was significantly upregulated in EC tissues compared to normal endometrial tissues (Fig. 4F). the upregulation is most pronounced in the high copy number (CNhigh) subgroup with poor prognosis. (Fig. 4G). Moreover, analysis of TCGA data and our clinical cohort showed that ACSS2 expression is upregulated in high-grade (G3) EC (Fig. 4H, I). This collectively suggests that ACSS2 is a potential molecule involved in the invasive progression of EC. Interestingly, knocking down *ACSS2* with siRNA in the cell model of long-term PS-NPs exposure attenuated the proliferation, migration, and invasion capacity of EC cells and enhanced their apoptosis (Fig. 4J–M). PS-NPs promote the progression of EC by mediating the upregulation of *ACSS2*.

### ACSS2 promotes *PLA2G3* acetylation and thus induces metabolic reprogramming in EC cells

**Fig. 5:**
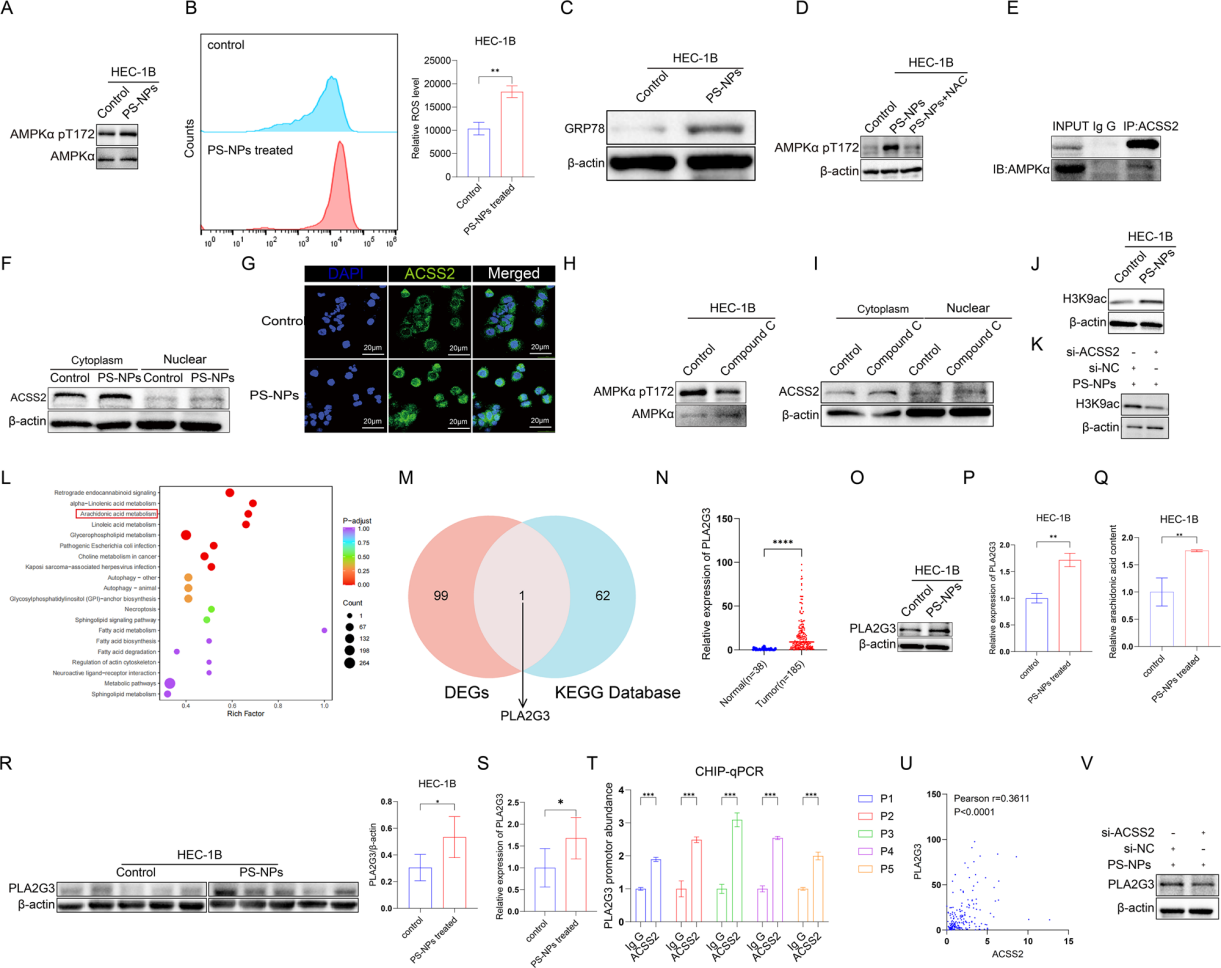
ACSS2 promotes *PLA2G3* acetylation and thus induces metabolic reprogramming in EC cells. Exposure to PS-NPs increases AMPKα phosphorylation, intracellular ROS, and ER stress levels (**A**–**C**). The ROS scavenger NAC can downregulate AMPK phosphorylation (**D**). Co-IP confirms the interaction between AMPK and ACSS2 (**E**). PS-NPs promote ACSS2 expression and nuclear translocation, which can be inhibited by the AMPK inhibitor Compound C (**F**–**I**). PS-NPs upregulate histone acetylation, and this effect depends on ACSS2 (**J**, **K**). Lipid-targeted metabolomics shows significant enrichment of the arachidonic acid (AA) metabolic pathway (**L**). Cross-analysis identifies the key gene PLA2G3 in the AA pathway (**M**). PLA2G3 is significantly upregulated in EC tissues (**N**). PS-NPs upregulate PLA2G3 expression and promote AA production cell model (**O**–**Q**). Mice that ingested PS-NPs showed increased expression of PLA2G3 mRNA and protein levels in their tumor tissues (**R**, **S**). ChIP experiments show that ACSS2 can bind to the PLA2G3 promoter (**T**). The mRNA expression levels of ACSS2 and PLA2G3 are significantly positively correlated (*r* = 0.3611, *p* < 0.0001) (**U**). Knocking down ACSS2 reduces PLA2G3 protein levels (**V**). Each experiment was performed with at least three biological replicates. For two-group comparisons, a t-test was applied. For comparisons across multiple groups, one-way or two-way ANOVA was employed. Values are expressed as mean ± SD. *p ≤ 0.05; **p ≤ 0.01; ***p ≤ 0.001; ****p ≤ 0.0001.

ACSS2 catalyzes the formation of acetyl CoA from acetic acid [43]. ACSS2 can be activated via adenosine 5’-monophosphate-activated protein kinase (AMPK) phosphorylation. Subsequently, activated ACSS2 enters the nucleus, binds to transcription factor EB, and translocates to the promoter regions of lysosomal and autophagy genes to mediate histone acetylation and regulate their transcription [44]. MP/NP can lead to an increase in intracellular ROS and endoplasmic reticulum stress [45–47]. Activation of AMPK is usually closely associated with ROS and endoplasmic reticulum stress [48, 49]. Lin et al. found that after knocking down *ACSS2*. NMDAR, and AMPAR showed low levels of histone acetylation within the promoters of these genes [50]. Meanwhile, Mews et al. discovered the upregulation of nuclear ACSS2 expression in differentiated neurons and the consequent increase in the expression of neuronal genes near sites of elevated histone acetylation [51]. In the present study, western blot assays revealed an increase in the phosphorylation of AMPKα after treatment with PS-NPs (Fig. 5A). We found an increase in intracellular ROS and the occurrence of endoplasmic reticulum stress (Fig. 5B, C). We treated the cells exposed to PS-NPs with the ROS scavenger N-acetylcysteine(NAC) and the ER stress alleviator 4-phenylbutyric acid(4-PBA). We found that treatment with NAC downregulated the phosphorylation level of AMPK (Fig. 5D), whereas treatment with 4-PBA did not change the phosphorylation level of AMPKα (Supplementary Figure 4A).

Furthermore, CO-IP results suggested that AMPK can bind to and interact with ACSS2 (Fig. 5E). Nucleoplasmic separation and immunofluorescence experiments demonstrated that exposure to PS-NPs could upregulate the expression of ACSS2 and increase its translocation to the nucleus (Fig. 5F, G). After AMPK inhibition using complex C, an AMPK inhibitor, the entry of ACSS2 into the nucleus appeared to be reduced (Fig. 5H, I). Additionally, we found that intracellular histone acetylation was upregulated after PS-NP exposure, but this effect was attenuated after *ACSS2* knockdown (Fig. 5J, K). These results suggest that PS-NPs can promote the translocation of ACSS2 to the nucleus by increasing ROS-induced AMPK activation of ACSS2 in cells.

ACSS2 regulates genes involved in lipid metabolism, thus affecting lipid production [52]. EC is a metabolic disease, and lipid metabolism plays a particularly important role in its development. Therefore, we performed targeted metabolomics analysis, focusing on lipids. Subsequently, the Kyoto Encyclopedia of Genes and Genomes (KEGG) pathway enrichment analysis revealed the significant enrichment of the arachidonic acid (AA) metabolic pathway (Fig. 5L). By comparing the top 100 genes in the DEGs identified via RNA-seq and the genes associated with the AA metabolic pathway in the KEGG database, we identified a common gene, *PLA2G3*, which encodes secretory phospholipase A2 (Fig. 5M). This enzyme hydrolyzes phospholipid molecules in cell membranes, releasing AA. Analysis of both the TCGA cohort and our center’s cohort consistently revealed that PLA2G3 is significantly upregulated in EC (Fig. 5N and Supplementary Figure 4B). We examined *PLA2G3* expression in PS-NP exposure models and found that PS-NPs induce the upregulation of *PLA2G3* mRNA and PLA2G3 protein levels, as well as promote the production of AA (Fig. 5O–Q). Mice treated with PS-NPs also exhibited upregulation of both *PLA2G3* mRNA and PLA2G3 protein levels(Fig. 5R, S). Moreover, ChIP results demonstrated that ACSS2 could occupy the promoter region of *PLA2G3* (Fig. 5T). In the analysis of clinical samples, we further found a significant positive correlation between the mRNA expression levels of *ACSS2* and *PLA2G3* (*r* = 0.3611, *p* < 0.0001) (Fig. 5U). And *ACSS2* knockdown led to the downregulation of and PLA2G3 protein levels (Fig. 5V). Therefore, the findings indicated that the accumulation of PS-NPs induces the metabolic reprogramming of AA through altered *ACSS2* expression.

### PS-NPs promote EC invasion and migration through the metabolic reprogramming of AA

**Fig. 6:**
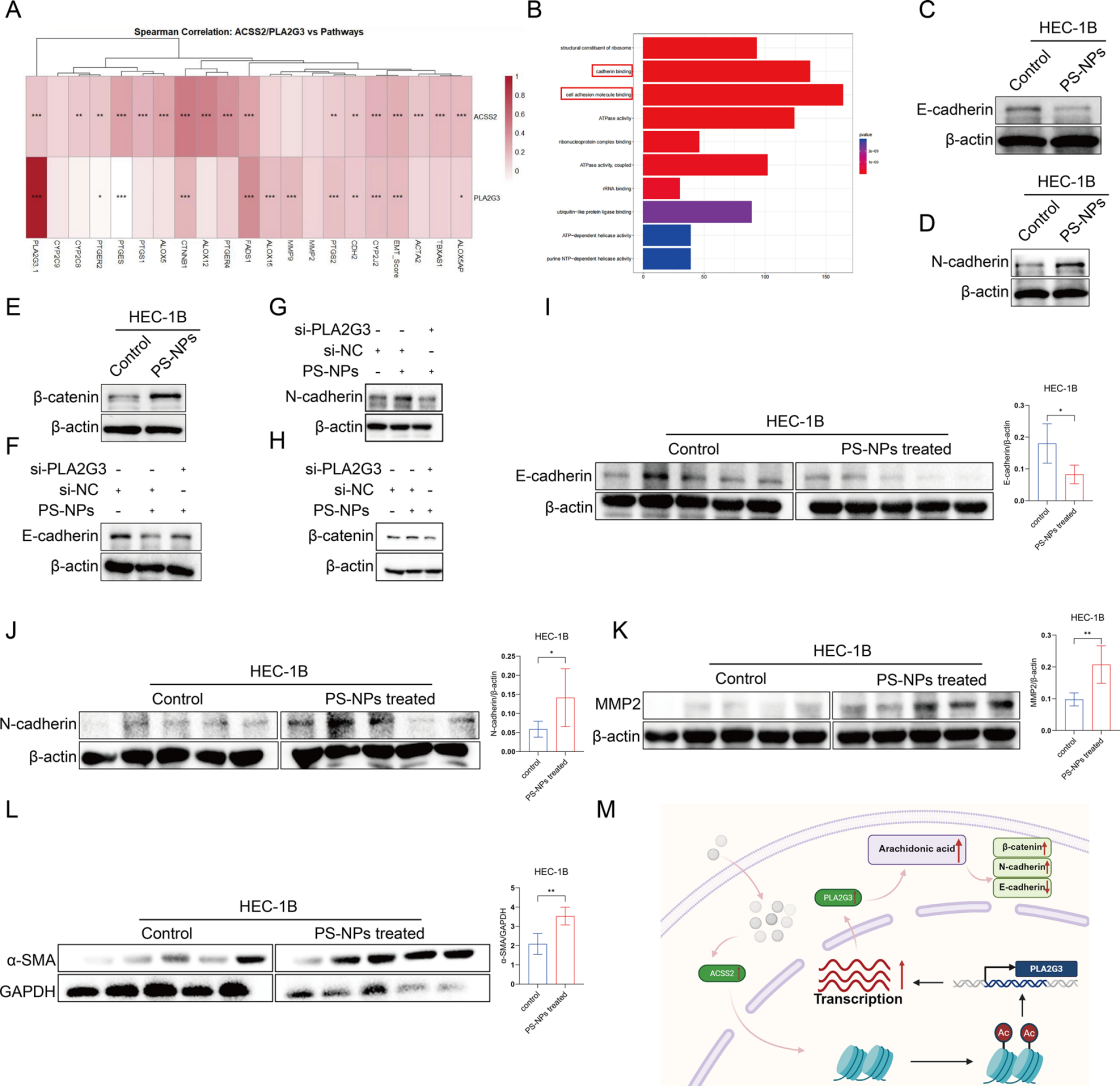
PS-NPs promote EC invasion and migration through the metabolic reprogramming of AA. ACSS2 is closely associated with PLA2G3, the AA pathway, and the EMT process (**A**). RNA-seq analysis shows that differentially expressed genes induced by PS-NPs are significantly enriched in functions such as cell adhesion molecule binding (**B**). Exposure to PS-NPs leads to downregulation of E-cadherin and upregulation of N-cadherin and β-catenin in HEC-1B cells (**C**–**E**). Knockdown of PLA2G3 can reverse the expression changes of the above EMT markers (**F**–**H**). In tumor-bearing mice tissues treated with PS-NPs, E-cadherin is downregulated, while mesenchymal markers N-cadherin, α-SMA, and MMP2 are upregulated (**I**–**L**). PS-NPs promote EC development through ACSS2-mediated reprogramming of arachidonic acid metabolism (**M**), Original figure created with BioRender.com. Each experiment was performed with at least three biological replicates. For two-group comparisons, a *t*-test was applied. For comparisons across multiple groups, one-way or two-way ANOVA was employed. Values are expressed as mean ± SD. *p ≤ 0.05; **p ≤ 0.01; ***p ≤ 0.001; ****p ≤ 0.0001.

Gómez-Valenzuela et al. demonstrated that AA promotes the epithelial-mesenchymal transition (EMT) in tumor cells by converting COX2 to prostaglandin E2 (PGE2) [53]. The TCGA database shows that *ACSS2* and *PLA2G3* are widely and significantly positively correlated with several key genes in the AA metabolic pathway as well as EMT scores. This suggests that ACSS2 and PLA2G3 are closely associated with the activation of the AA pathway and the EMT process (Fig. 6A). RNA-seq data showed that the DEGs induced by PS-NP exposure were significantly enriched in molecular functions such as cell adhesion molecule binding and cadherin binding (Fig. 6B). Our experimental findings showed that exposure to PS-NPs resulted in the significant downregulation of E-cadherin in HEC-1B cells, as well as the significant upregulation of N-cadherin and β-catenin (Fig. 6C–E). Meanwhile, the knockdown of *PLA2G3* upregulated E-cadherin, and downregulated N-cadherin and β-catenin (Fig. 6F–H). In tumor tissues of mice treated with PS-NPs, EMT markers changed: the epithelial marker E-cadherin was downregulated, while the expression of mesenchymal markers N-cadherin, α-SMA, and MMP2 was upregulated (Fig. 6I–L).

Collectively, the findings suggested that *PLA2G3* mediates the altered expression of cell adhesion molecules and cadherin binding-related genes after PS-NP exposure by regulating the production of AA, thus inducing EMT. These changes may promote the invasion and metastasis of tumor cells (Fig. 6M).

## Discussion

MP and NPs have been detected in human saliva, blood, digestive tissues, and endometrial tissues [11–19, 31]. Hence, their health hazards warrant urgent investigation. So far, the risk of PS-NPs promoting the progression of EC has not been explored. In this study, we demonstrated that PS-NPs show effective uptake and accumulation in both EC cells and organoids. The cellular uptake of PS-NPs appears to be time-dependent, with fluorescent signals intensifying over 72 h of exposure and eventually being detected in nearly all cells. In contrast, the uptake of PS-NPs in EC organoids seems slower, likely due to the sequestering effect of stromal gels, with signals progressively increasing over 13 days of treatment. These findings highlight the ability of PS-NPs to penetrate and accumulate in EC cells and organoids, suggesting their potential impact on EC.

PS-NPs are present in the food chain and can be transported to endometrial tissue through the bloodstream [16, 31, 54]. In Chen et al.’s study, microfibers containing 2/3 MPs were found much more frequently in tumors from lung cancer patients than in healthy tissues, indicating a potential relationship between MP exposure and carcinogenesis [55]. Meanwhile, using radioisotope-labeled and fluorescently tagged PS, Kim et al. discovered the accumulation of PS in the stomach of mice. Moreover, in a 4-week PS-exposed NCI-N87 mouse model, the researchers found that PS exposure induces cancer stemness and multidrug resistance, and mice injected with PS-MP-treated cells showed faster tumor growth and lower survival rates than the control group [28]. In our study, orally exposed PS-NPs can reach distant organs and affect tumor growth. And the addition of 40 mg/L PS-NPs to drinking water accelerated tumor growth in mice. These findings were consistent with previous reports showing that PS-MP/NP exposure accelerates carcinogenesis in mammals [28, 29]. Our findings indicate that long-term exposure to PS-NPs promotes EC progression by significantly enhancing the proliferation, invasion, and migration of tumor cells while reducing their apoptosis. These results suggest that prolonged exposure to PS-NPs may serve as a potential risk factor for cancer development.

To further explore the mechanism by which PS-NPs affect EC progression, we performed RNA-seq. We found that the *ACSS2* gene was upregulated in EC after prolonged PS-NP exposure, and the qPCR results were validated using western blot assays. Notably, we observed decreased tumor cell proliferation, invasion, and migration and increased tumor cell apoptosis after knocking down *ACSS2*. Recent studies have shown that ACSS2 can be activated via AMPK phosphorylation. Subsequently, it translocates to the nucleus, binds to TFEB, localizes to gene promoter regions, alters histone acetylation, and thereby regulates gene transcription [44, 50, 51]. Our results show that exposure to PS-NPs promotes AMPK phosphorylation via ROS. Phosphorylated AMPK interacts with ACSS2, resulting in increased nuclear translocation of ACSS2. Here, ACSS2 upregulates histone acetylation, and knocking down *ACSS2* can attenuate histone acetylation. Furthermore, after inhibiting AMPK with an AMPK phosphorylation inhibitor, the entry of ACSS2 into the nucleus can be reduced. These findings suggest that phosphorylated AMPK activates ACSS2, which translocates to the nucleus, thereby increasing histone acetylation.

EC is a metabolic disease [32–35]. In particular, lipid metabolism plays a key role in the development of EC. Hence, we performed targeted lipid metabolomics analysis in this study. Subsequently, KEGG enrichment analysis of differential metabolites revealed significant enrichment for the AA pathway. Hence, we combined the DEGs identified via RNA-seq with the genes related to the AA metabolism pathway and found that *PLA2G3* was significantly upregulated after exposure to PS-NPs. Subsequently, ChIP results revealed the significant enrichment of ACSS2 within the promoter region of *PLA2G3*. And after knocking down *ACSS2*, *PLA2G3* expression was found to be downregulated. These results suggest that ACS22 may promote histone acetylation within the promoter region of *PLA2G3* and thus regulate the transcription of this gene. However, whether ACSS2 is enriched at the *PLA2G3* promoter region marked by H3K9ac remains to be verified by ChIP in future. *PLA2G3* encodes secretory phospholipase A2, which hydrolyzes phospholipid molecules in cell membranes, releasing AA. Thus, we examined AA levels after exposure to PS-NPs and found that the levels were significantly elevated. However, the levels of AA were reduced when *PLA2G3* was knocked down. AA can generate PGE2 via the COX2 pathway, leading to EMT in cancer cells [53]. The DEGs were significantly enriched for molecular functions such as cell adhesion molecule binding and cadherin binding. Moreover, we found changes in EMT-related markers after exposure to PS-NPs, suggesting that PS-NPs promote tumor cell proliferation, migration, and invasion.

Through in vitro experiments, our study demonstrated that PS-NPs can be absorbed and accumulated by EC tumor cells and organoids. Through in vivo experiments, we found that PS-NPs can also be ingested by mice through drinking water and accumulate in tumors, thereby promoting tumor development. Additionally, we elucidated the specific mechanisms involved in this carcinogenic process and the role of metabolic alterations. Our findings highlight that PS-NP exposure is a new threat and risk factor for EC development and provide new therapeutic targets for mitigating the effect of PS-NPs on tumor progression.

## Conclusion

Here, we demonstrated that PS-NPs are taken up by EC cells and organoids and accumulate within them. In vitro, exposure to PS-NPs promotes the proliferation, migration, and invasion capacity of EC cells. Meanwhile, exposure to PS-NPs via drinking water increases tumor sizes in mouse models in vivo. On a mechanistic level, PS-NPs activate *ACSS2*, which may facilitate an epigenetic state at the *PLA2G3* promoter consistent with enhanced histone acetylation, thereby upregulating *PLA2G3* expression and driving metabolic reprogramming of the AA pathway. Subsequently, the increased production of AA and PGE2 facilitates the progression of EC. This study reveals the genetic, signaling pathway, and molecular alterations induced by PS-NPs in EC cells, which could serve as early biomarkers and research candidates for studying and mitigating the potential impact of PS-NPs on EC.

## Material and methods

### Cell culture and exposure to PS-NPs

The HEC-1B cells were purchased from Jennio Biotech (Guangzhou, China), and cell line Ishikawa was obtained from the China Center for Type Culture Collection (CCTCC, Wuhan, China). The cells were cultured in RPMI-1640 medium containing 10% fetal bovine serum (FBS) and 1% penicillin/streptomycin. The environment of the incubator was 37 °C, 5% CO2 and humidity. When the cells were more than 80% confluent, the old medium was removed and washed with phosphate-buffered saline (PBS). Then cells were digested with 0.25% trypsin-ethylenediaminetetraacetic acid (trypsin-EDTA) and centrifuged after gently blowing to dislodge the cells to form a single cell suspension. Finally, the cells were resuspended with medium and transferred into new cell culture dishes containing fresh medium. Fresh medium containing 15 mg/L PS-NPs was changed daily for one month. The treated cells were used for subsequent experiments.

### PS-NPs characterization

PS-NPs were dispersed in ultrapure water. The hydrodynamic diameter and Zeta potential were measured using a Malvern Zetasizer (Nano-ZS, Malvern Instruments, England).

### Setting and participants

The study was approved by the Research Ethics Committee of the Third Affiliated Hospital of Guangzhou Medical University (NO: 2020066), and informed consent was obtained from all patients. This study included adult patients (age ≥ 18 years) who had not received prior treatment and were histologically confirmed to have surgically resectable EC. All participants underwent surgery at the Third Affiliated Hospital of Guangzhou Medical University between April 22, 2023, and March 30, 2025. Tumor tissues from 4 independent patients were used for organoid culture in this study. Among the 4 attempted samples, organoid lines were successfully established and stably passaged in 3 cases, with a success rate of 75%.

### EC tissue collection and organoid construction

The extraction of EC organoids begins with freshly surgically removed tumor tissue. After preserving the tissue in a protective solution, it is washed with PBS to remove normal and necrotic parts, then mechanically cut into 1–2 mm³ fragments. Subsequently, the tissue fragments are digested at 37 °C with a digestion solution specific for EC organoids for 1–2 h with shaking. After digestion, the solution is pipetted to disperse cells, filtered through a 100 µm mesh, and centrifuged for washing to obtain a cell pellet. Finally, the cell pellet is resuspended in a 2:3 mixture of organoid culture medium (Guangzhou Orgen Biotech Co., Ltd, China) and Matrigel (Corning, New York, USA), seeded into pre-warmed culture plates, overlaid with culture medium after solidification at 37 °C, and placed in a humidified incubator at 37 °C with 5% CO_2_ to start organoid culture. Organoids are routinely passaged mechanically every 3–7 days. All key organoid experiments were conducted with at least 3 independent biological replicate passages. The passage numbers of organoids used in the experiments ranged from P3 to P10.

### Xenograft experiments and PS-NP exposure

All animal experiments were conducted in accordance with the National Institutes of Health guidelines for laboratory animals. And the animal experiments received ethical approval from the Guangdong Medical Laboratory Animal Center (No: B202411-6). Female BALB/c nude mice, aged 4–6 weeks, were obtained from the Guangdong Laboratory Animal Center (Foshan, Guangdong). Subsequently, HEC-1B cells (6 × 10⁶) were administered via subcutaneous injection into the right flank of the mice. After the tumors became palpable, all mice were randomly divided into two groups. The control group was provided with PS-NP-free drinking water (0 mg/L) for 46 days, while the treatment group received water containing 40 mg/L PS-NPs over the same period. Tumor growth and body weight were monitored at 3-day intervals. Following the experimental period, mice were humanely sacrificed by cervical dislocation. Excised tumors were photographed, weighed, and sectioned into three equal parts for different analyses: RNA isolation, fixation in formalin followed by paraffin embedding, or snap-freezing in liquid nitrogen. Tumor volume (mm3) was calculated as = *a* × *b*^2^ × 0.5 (*a* is the longest diameter, *b* is the shortest diameter, and 0.5 is a constant for calculating the volume of the ellipsoid).

### Uptake of PS-NPs by HEC-1B cells and EC organoids

To begin with, 3 × 10⁵ HEC-1B cells were placed into six - well chamber slides and left to adhere. The day after, the cells were incubated at a temperature of 37 °C within a medium that had been incorporated with fluorescently tagged PS-NPs. The incubation periods were set at 0, 24, 48, and 72 h, respectively. After that, the cells underwent washing with PBS in order to get rid of any PS-NPs that were clinging to the cell surface. Next, the living cells were fixed using 4% paraformaldehyde for a duration of 15 min, and the cell nuclei were then stained by 4′,6 - diamidino - 2 - phenylindole (DAPI). When using a confocal microscope, the fluorescently labeled PS-NPs, which showed red fluorescence, were activated by a laser at a wavelength of 535 nm, and the resulting emitted signal was captured at 610 nm. At the same time, DAPI, which presented blue fluorescence, was excited at 405 nm, and the emitted signal from it was detected at 455 nm. Finally, confocal laser scanning microscopy (CLSM) was utilized to gather the images.

### Cell Counting Kit-8 (CCK-8) assay

First, 3000 cells were added to each well of a 96-well plate. After the cells fully adhered to the wells, cell viability was determined after 0, 24, 48, and 72 h of incubation with PS-NPs using the CCK-8 kit (Yeasen, Shanghai, China).

### Wound healing assay

HEC-1B and Ishikawa cells incubated with or without PS-NPs for 4 weeks were added to 6-well plates at a density of 1 × 10^6^ cells/well. When the cells successfully formed a monolayer, a 200 μL pipette tip was used to create a scratch across the diameter of each monolayer. Images were obtained under a microscope at 0, 24, and 48 h after inoculation.

### Transwell assay

Following 4-week incubation with or without PS-NPs, cells were seeded into the Transwell insert. The lower compartment had been pre-coated with Matrigel, with 600 μL of 10% FBS-supplemented RPMI-1640 medium. The upper chamber contained 200 μL of serum-free RPMI-1640 medium. Following 48 h of incubation (37 °C, 5% CO₂), cells were fixed and stained with crystal violet solution. Non-migratory cells remaining on the upper membrane surface were carefully removed using cotton swabs. Microscopic examination was conducted at 20× magnification to visualize the stained cells, with invasive potential quantified by enumerating cells in three randomly selected visual fields per experimental condition.

### Immunofluorescence assays

First, cells were inoculated into 6-well plates fitted with coverslips (3 × 10^5^ cells per well). After 48 h of incubation, the cells were fixed in 4% paraformaldehyde to maintain their morphology and immobilize the antigen. They were then washed three times with PBS for 5 min each. The cells were then treated with Triton X-100 (Beyotime, Shanghai, China) to increase the permeability of the cell membrane. The cells were then washed three more times with PBS for 5 min each.Subsequently, after the cells were closed using 5% BSA, ACSS2 antibody (16087-1-AP, 1:200, Proteintech, Wuhan, China) was added and incubated at 4 °C overnight to ensure that the antibody could bind to the antigen sufficiently. The cells were then washed three times with 1× PBS-Tween 20 (PBST) buffer for 5 min each time. The cells were then incubated with fluorescently labeled secondary antibody (SA00001-1, SA00001-2, Proteintech, Wuhan, China) for 1.5 h at room temperature. The cells were washed three times with 1× PBST buffer for 5 min each. Finally, the nuclei were stained with DAPI, and the coverslips were mounted on slides for observation with CLSM.

### Quantitative polymerase chain reaction (qPCR) analysis

Total cellular RNA was isolated employing RNAisoPlus reagent (Takara, Shiga, Japan) following the standard protocol. Subsequently, 5 μg of purified RNA was reverse transcribed into complementary DNA using a commercial cDNA Synthesis Kit (Yeasen, Shanghai, China) as per the manufacturer’s guidelines. Quantitative PCR(qPCR) amplification was performed using Hieff® qPCR SYBR Green Master Mix on a QuantStudio 3 thermocycler (QuantStudio 3, Foster City, CA, USA). Relative expression levels of genes were calculated by the formula RQ = 2^−ΔΔCt^, and all reactions were performed in technical triplicate.

### Western blotting

Cells were pelleted by centrifugation and the supernatant was removed. After two PBS washes to eliminate residual serum proteins, pre-cooled RIPA lysis buffer supplemented with 1 mM PMSF was added. Cell lysis was performed on ice for 15 min, followed by protein quantification using a BCA assay kit (Beyotime, Shanghai, China). Electrophoresis was performed using polyacrylamide gels of appropriate concentrations matched to the expected molecular weights of the proteins of interest. Pre-stained protein ladders (ThermoFisher Scientific, USA) were run in parallel for molecular weight calibration. Following electrophoresis, PVDF membranes were methanol-activated prior to protein transfer. Subsequently, the membranes underwent blocking with 3% BSA solution at room temperature for 90 min. The primary antibody anti-ACSS2 (16087-1-AP, Proteintech, Wuhan, China), anti-PLA2G3 (A07886-1, BODYC, Wuhan, China), anti-AMPKa pT172 (4143, Cell Signaling Technology, Danvers, MA, USA), anti-GRP78 (11587-1-AP, Proteintech, Wuhan, China), anti-H3K9ac (29133-1-AP, Proteintech, Wuhan, China), anti-E-cadherin (20874-1-AP, Proteintech, Wuhan, China), anti-N-cadherin (22018-1-AP, Proteintech, Wuhan, China), anti-β-catenin (51067-2-AP, Proteintech, Wuhan, China), anti-α-SMA (AG8004, Beyotime, Shanghai, China), anti-MMP2 (10373-2-AP, Proteintech, Wuhan, China), anti-ER (21244-1-AP, Proteintech, Wuhan, China), anti-PR (25871-1-AP, Proteintech, Wuhan, China), anti-HOXA10 (26497-1-AP, Proteintech, Wuhan, China) were diluted with antibody diluent and incubated with the membrane gently shaking overnight at 4 °C. Following three 5-min TBST washes, the secondary antibody incubation was performed at room temperature for 90 min. The membrane was washed three more times with TBST for 5 min each time. Finally, the membrane was color developed and imaged with enhanced chemiluminescence reagent (ChemiDocTMXRS + , BIO-RAD, USA).

### Co-immunoprecipitation (CO-IP) assay

To collect protein samples, cells were lysed using an immunoprecipitation lysis buffer (Beyotime, Shanghai, China) containing 1 mM PMSF. Then, a primary antibody against the target protein was added. After 16 h of incubation to enable antibody-protein binding, Protein A/G Magnetic Beads were added to form a magnetic bead-antigen-antibody complex. This complex was separated from unbound proteins using a magnetic separation rack, and the proteins were eluted from the magnetic beads. Finally, the eluted proteins were used for western blot analysis to detect and identify proteins bound to the target protein.

### Chromatin immunoprecipitation (ChIP) assay

When the cell confluence reached 90%, the culture medium was replaced with a fresh complete medium containing 1% formaldehyde, and the cells were incubated at 37 °C for 10 min. After cross-linking and the fixation of intracellular protein and DNA complexes, ChIP was performed using the BeyChIP™ Enzyme catalyzed ChIP system (Beyotime, Shanghai, China) in line with the manufacturer’s instructions. Finally, the isolated DNA was used for qPCR analysis.

### Transcriptional analysis of HEC-1B cells after PS- NP exposure

Three samples each were collected from the control group and treated group. Transcriptome sequencing and analysis were conducted by Guangzhou Epiview Biotechnology Co., Ltd. (Guangzhou, China).

### Targeted metabolomics analysis of HEC-1B EC cells after PS-NP exposure

Twelve samples were collected and divided into two groups for comparative analysis. The lipid composition of the cells was detected by Metware Biotechnology (Wuhan, China) using a UPLC-MS/MSS platform.

### Data analysis

Results are presented as mean ± SD (standard deviation). Each experiment was performed with at least three biological replicates. For two-group comparisons, a *t*-test was applied. For comparisons across multiple groups, one-way or two-way ANOVA was employed. Statistical significance was set at *P < 0.05, **P < 0.01, ***P < 0.001, ****P < 0.0001. All statistical procedures were conducted with PRISM version 5.0 (GraphPad, California, USA).

## Supplementary information

Supplementary Information

original Western blot

## Data Availability

All data generated or analysed during this study are included in this published article. The data used in this study include the publicly accessible TCGA endometrial cancer dataset, as well as newly generated sequencing data from this study—the related raw sequence data have been publicly deposited in the NCBI Sequence Read Archive under accession number PRJNA1418129 (access link: https://www.ncbi.nlm.nih.gov/sra/PRJNA1418129). The metabolomics data involved in this study have been deposited in the MetaboLights database, with the study identifier MTBLS13850 (access URL: https://www.ebi.ac.uk/metabolights/MTBLS13850).
