# Supplementary material for: Polystyrene nanoparticles promote endometrial cancer development through the ACSS2-mediated reprogramming of arachidonic acid metabolism
Source: Cell Death Discov. 2026 Mar 26;12:189. doi: 10.1038/s41420-026-03071-5 (PMC13139601; doi:10.1038/s41420-026-03071-5)
Supplement: Supplementary file 1 — Supplementary Information [file 41420_2026_3071_MOESM1_ESM.pdf]

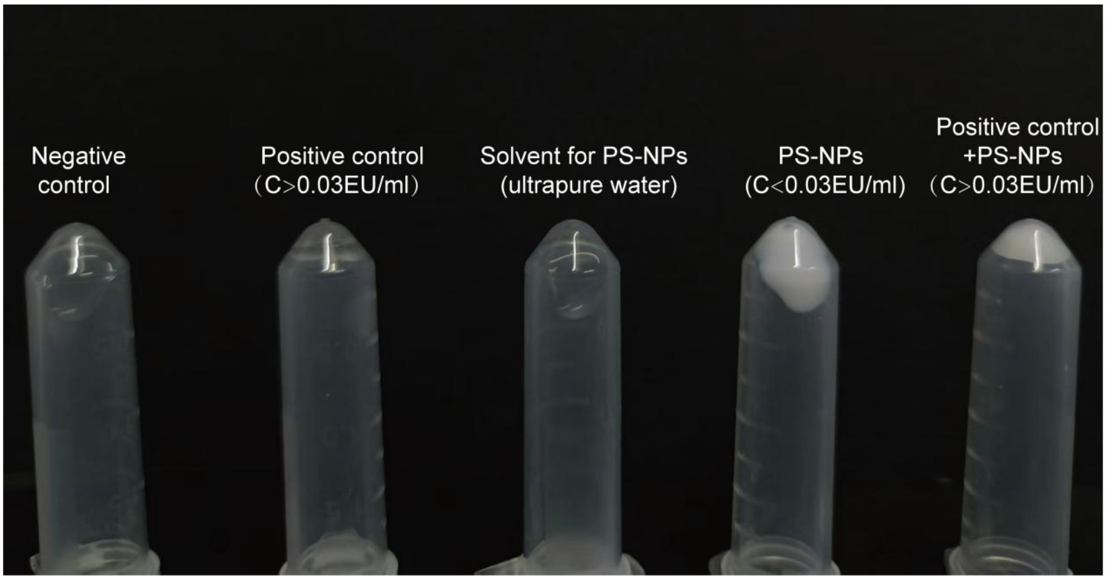

Supplementary Figure 1: The endotoxin level in PS-NPs was measured by the gel-clot LAL assay and was determined to be <0.030 EU/mL

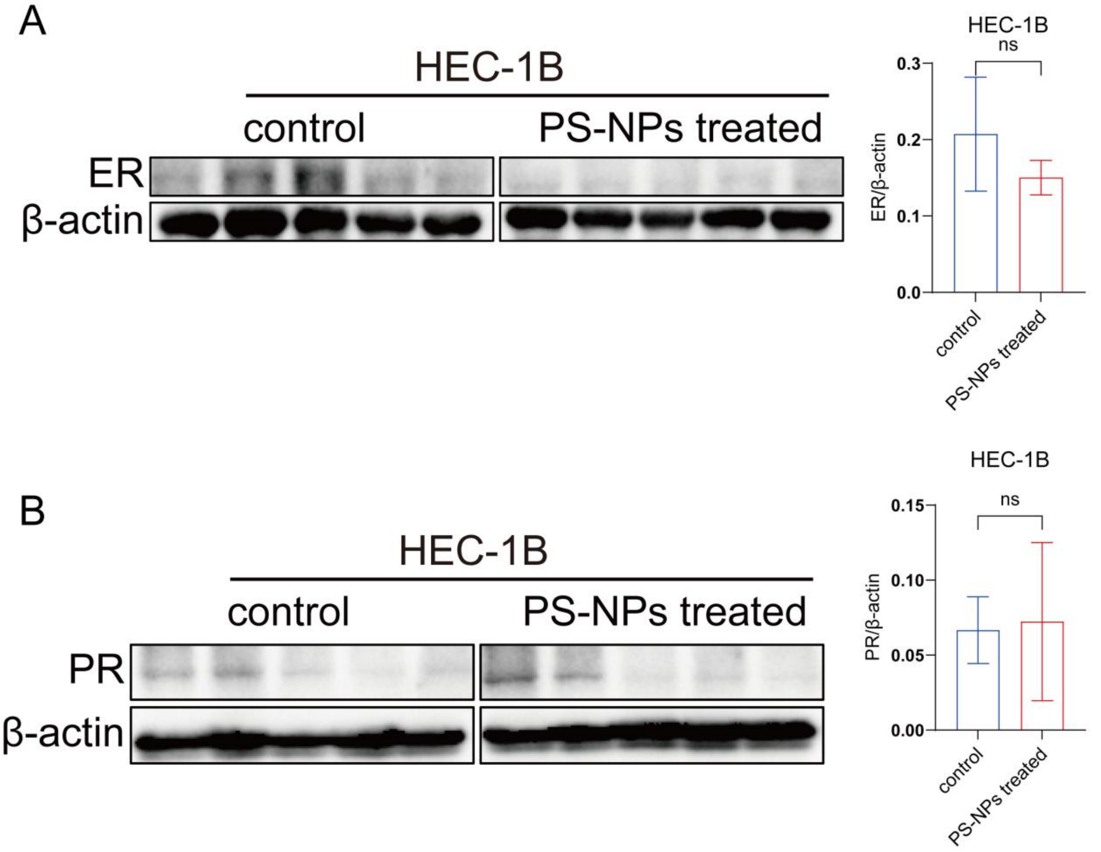

Supplementary Figure 2： There were no significant differences in the expression levels of estrogen receptor (ER) and progesterone receptor (PR) between the two groups

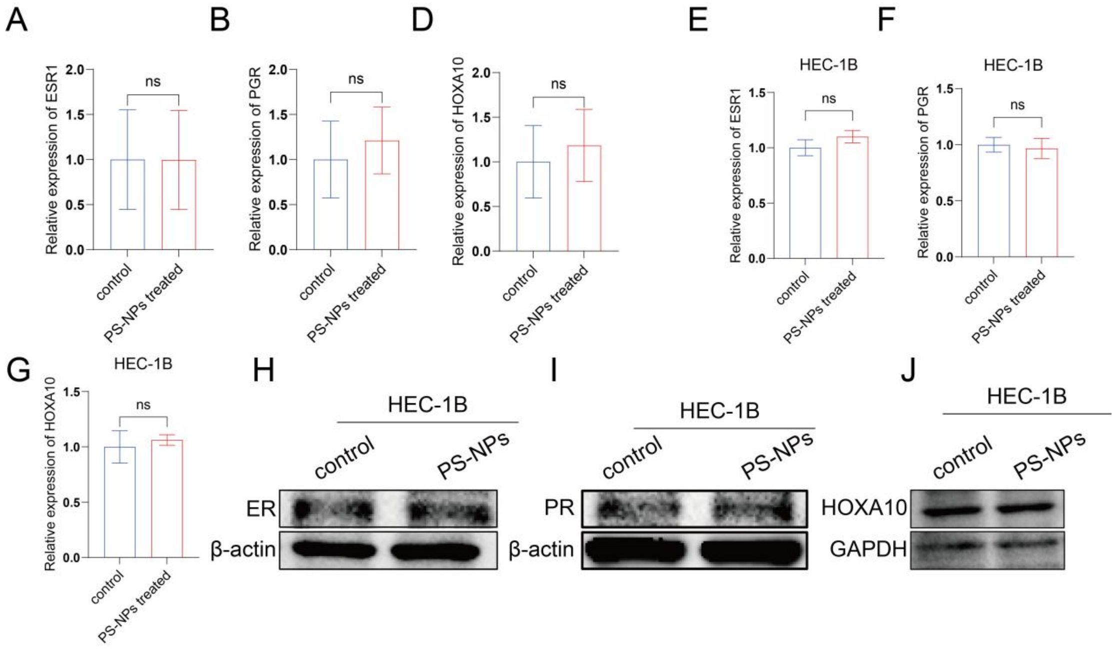

Supplementary Figure 3: PS-NPs exposure does not affect the expression of hormone-related genes (ESR1, PGR, HOXA10) in vitro and in vivo.

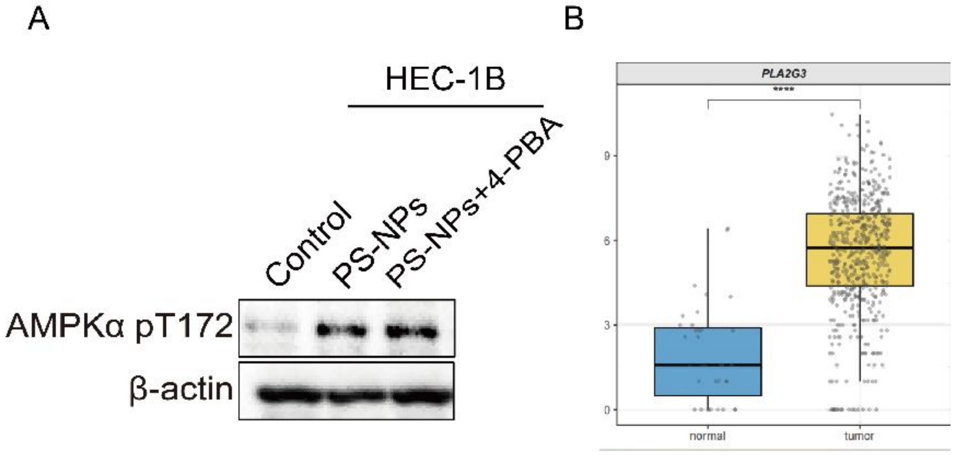

Supplementary Figure 4: Treatment with 4-PBA did not reverse the increased phosphorylation of AMPK caused by PS-NPs treatment (A). Analysis of TCGA data reveals that PLA2G3 expression is upregulated (B).
