# Supplementary material for: Polystyrene nanoparticles promote endometrial cancer development through the ACSS2-mediated reprogramming of arachidonic acid metabolism
Source: Cell Death Discov. 2026 Mar 26;12:189. doi: 10.1038/s41420-026-03071-5 (PMC13139601; doi:10.1038/s41420-026-03071-5)

HEC-1B control vs. HEC-1B PS-NPs treated (ACSS2)

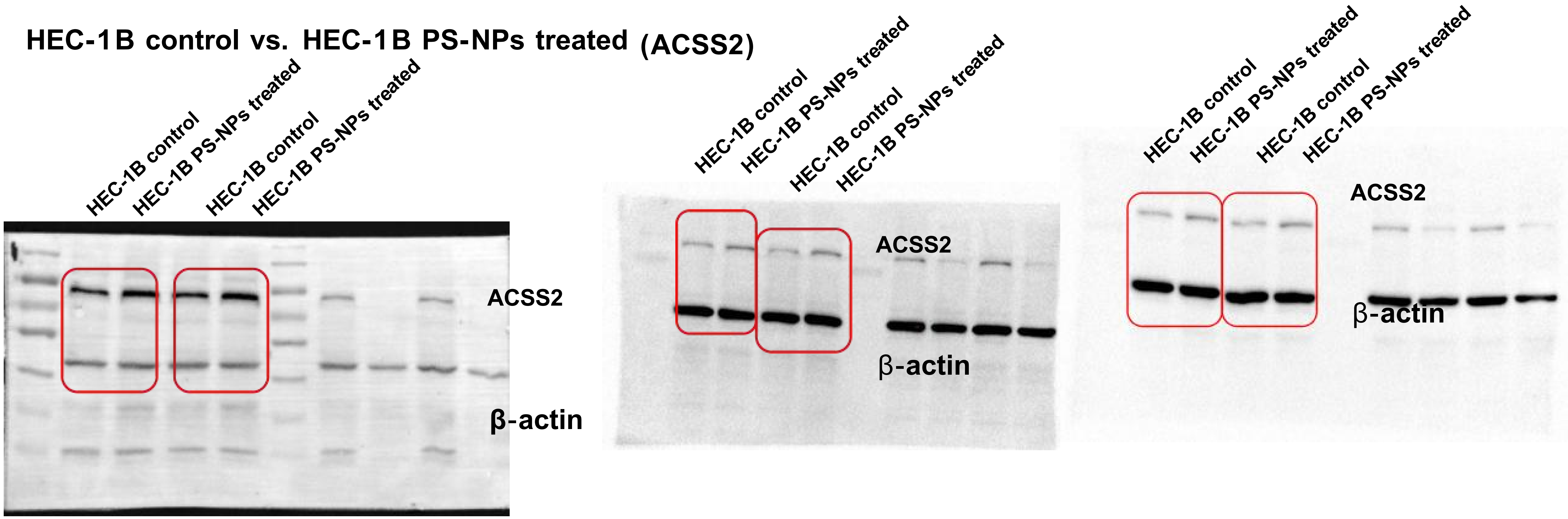

Ishikawa control vs. Ishikawa PS-NPs treated (ACSS2)

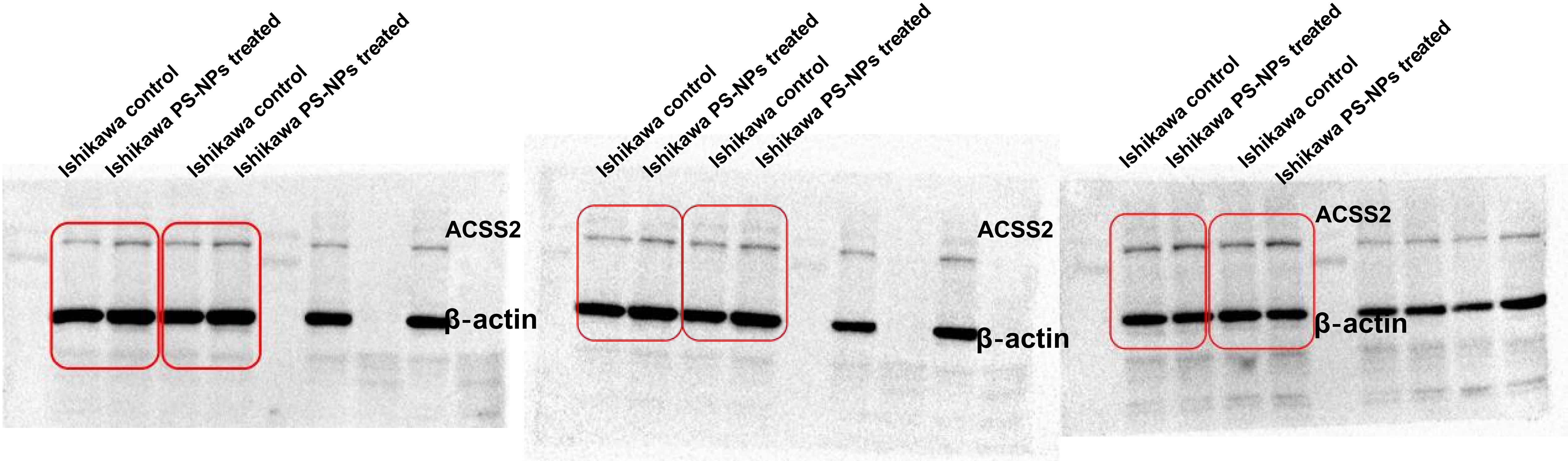

HEC-1B control vs. HEC-1B PS-NPs treated (ER)

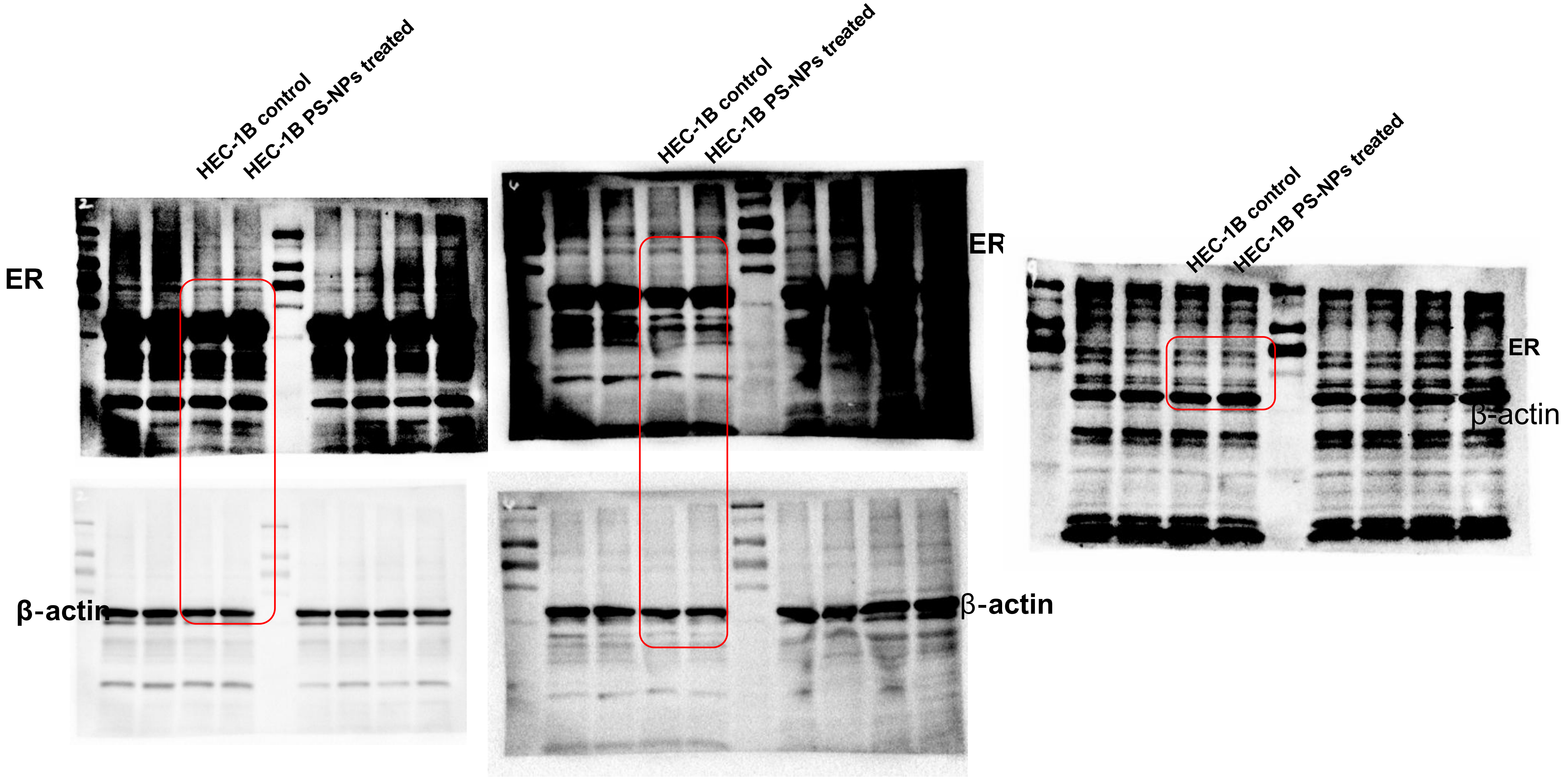

HEC-1B control vs. HEC-1B PS-NPs treated (PR)

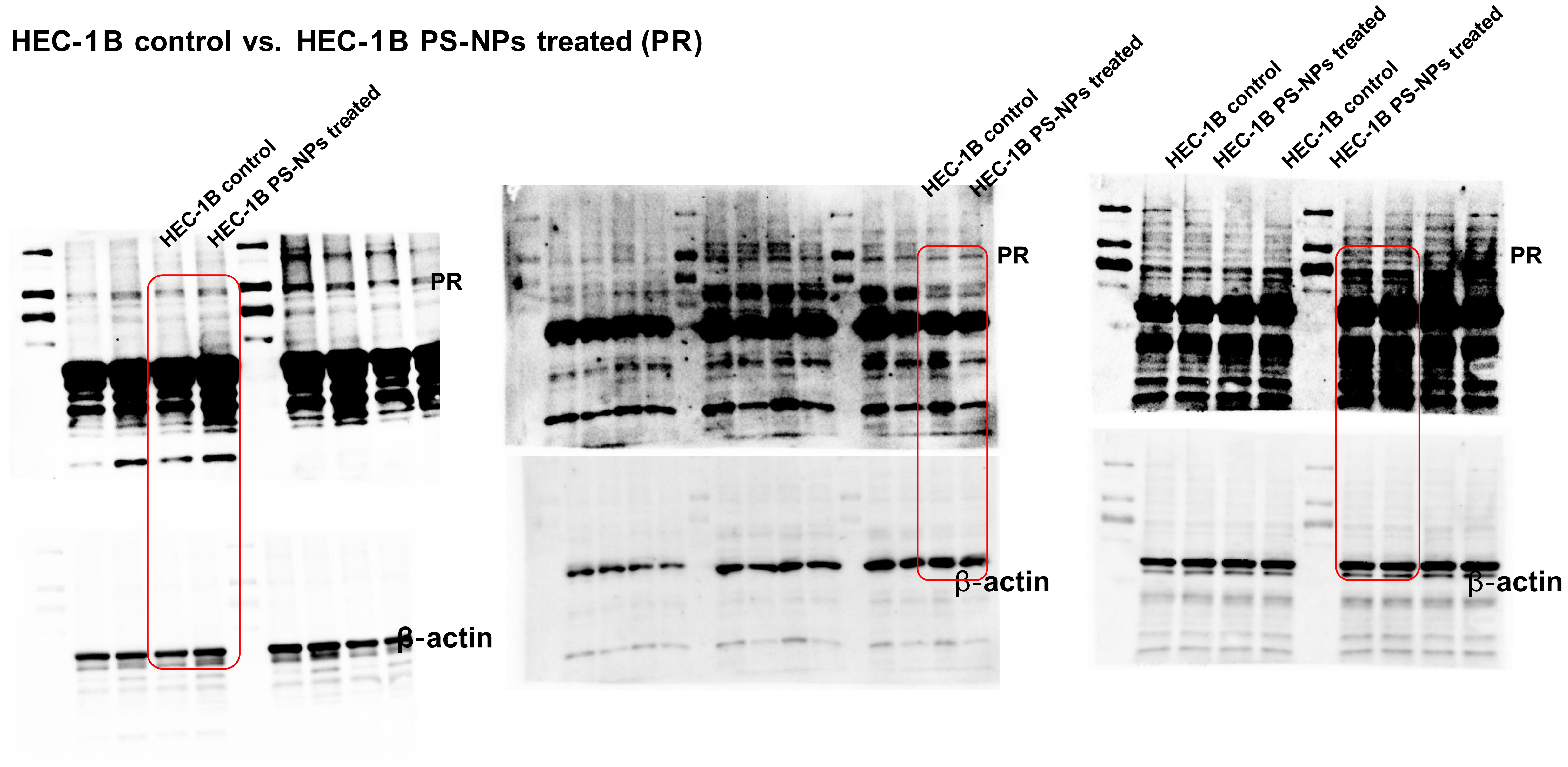

HEC-1B control vs. HEC-1B PS-NPs treated HOXA10

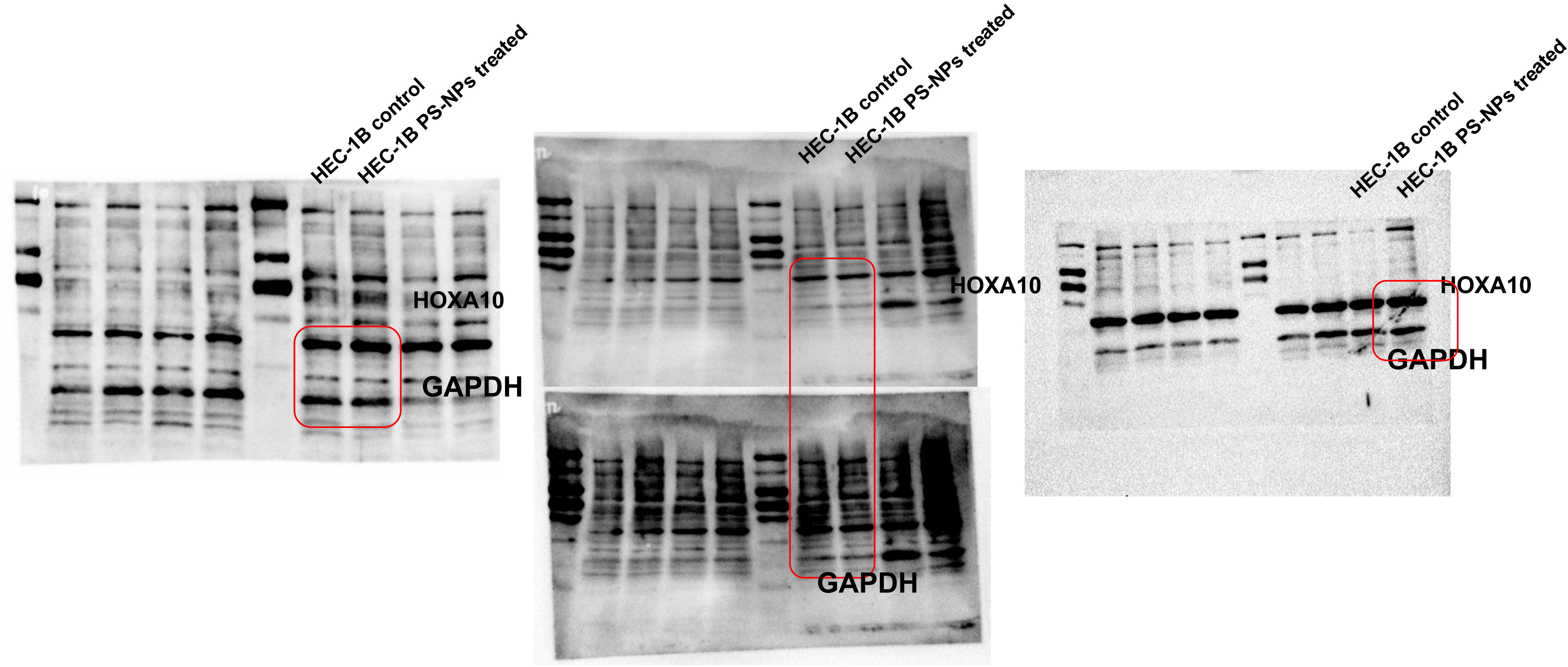

HEC-1B control vs. HEC-1B PS-NPs treated（GRP78）

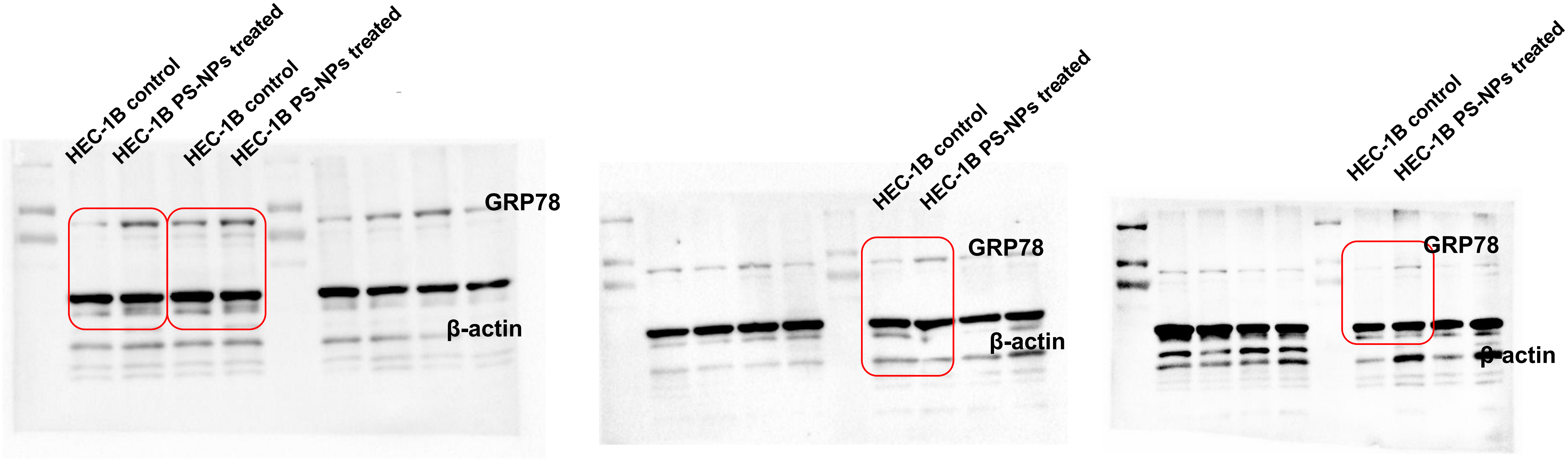

COIP IP：ACSS2 IB: AMPK

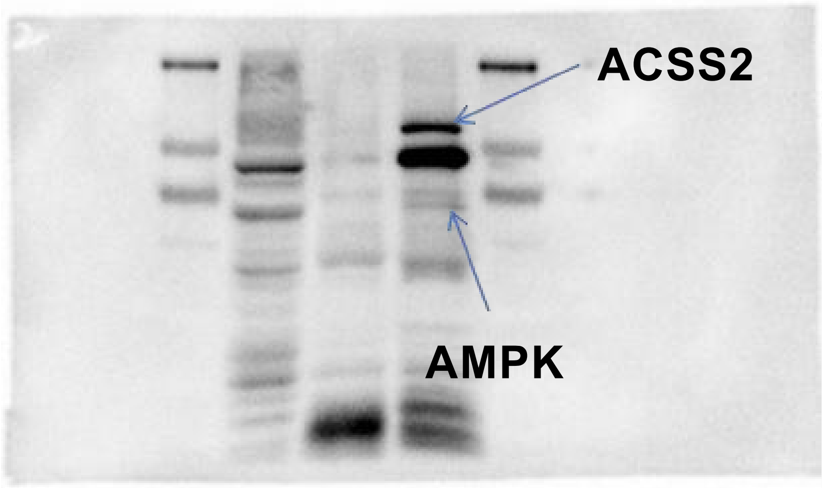

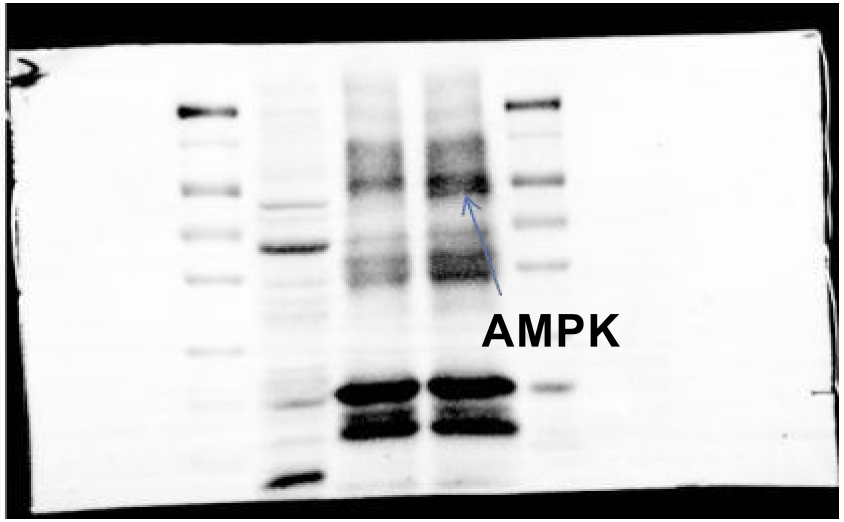

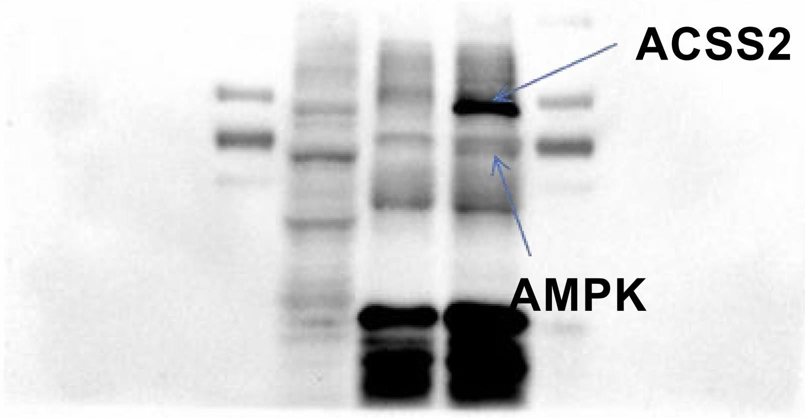

HEC-1B control vs. HEC-1B PS-NPs treated (AMPKα pT172)

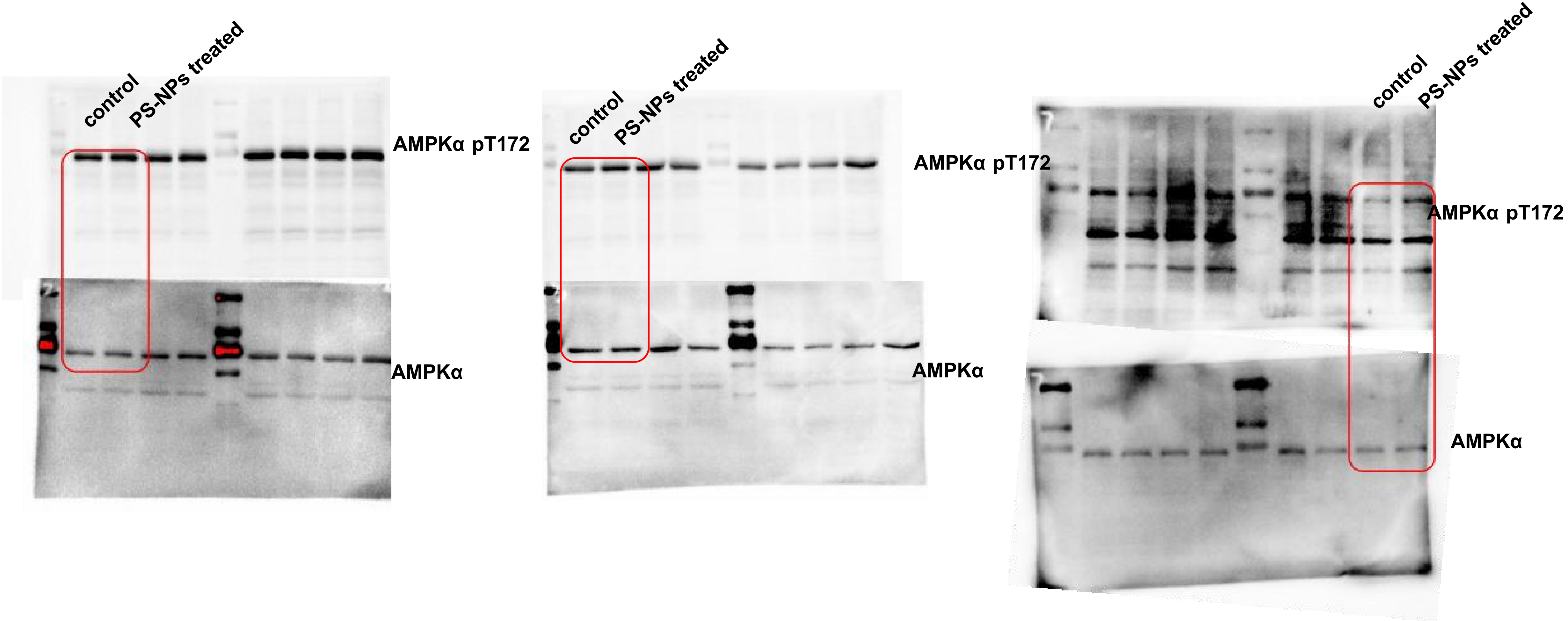

HEC-1B control vs. HEC-1B compound C (AMPKα pT172)

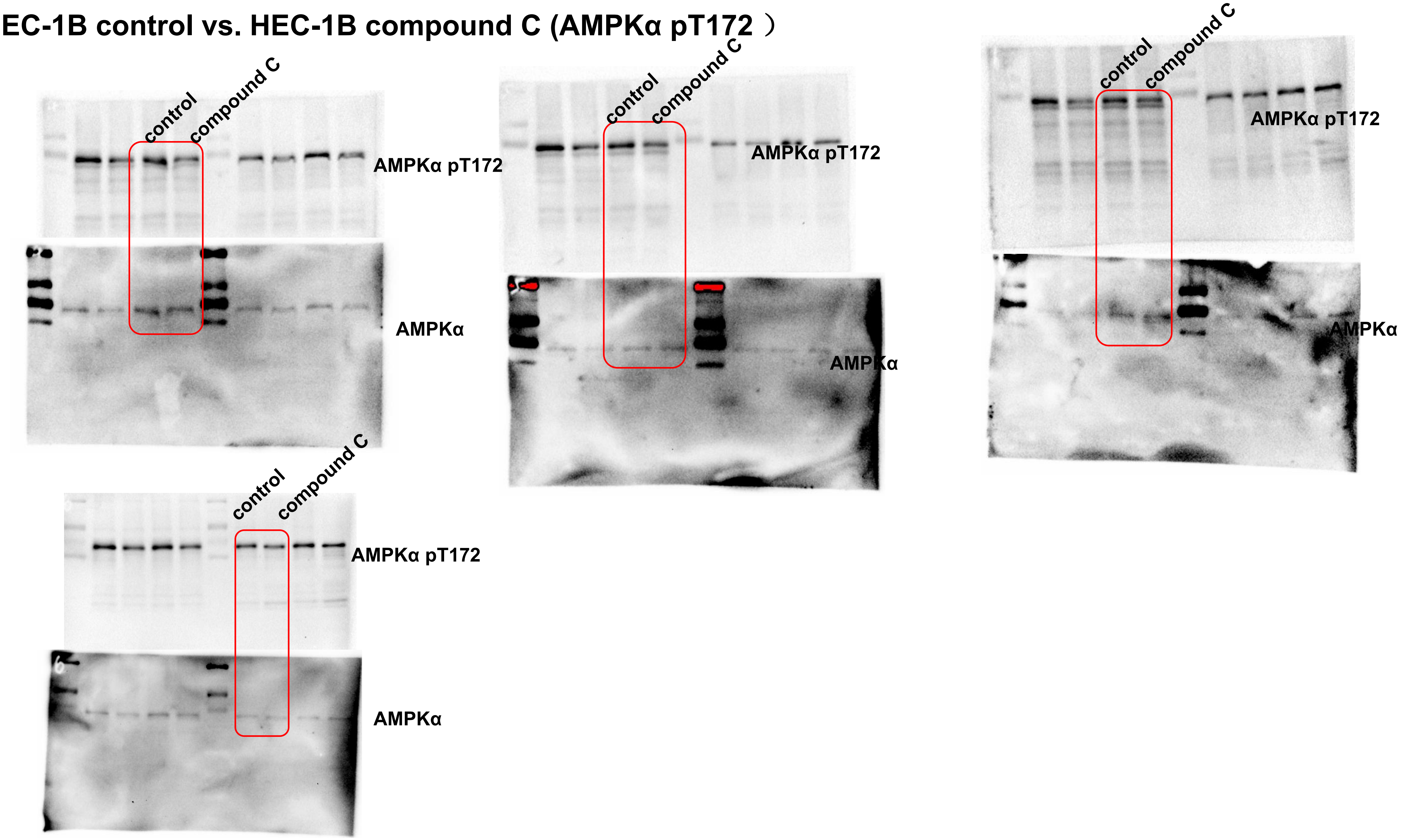

Nucleoplasmic separation HEC-1B control vs. HEC-1B PS-NPs treated (ACSS2)

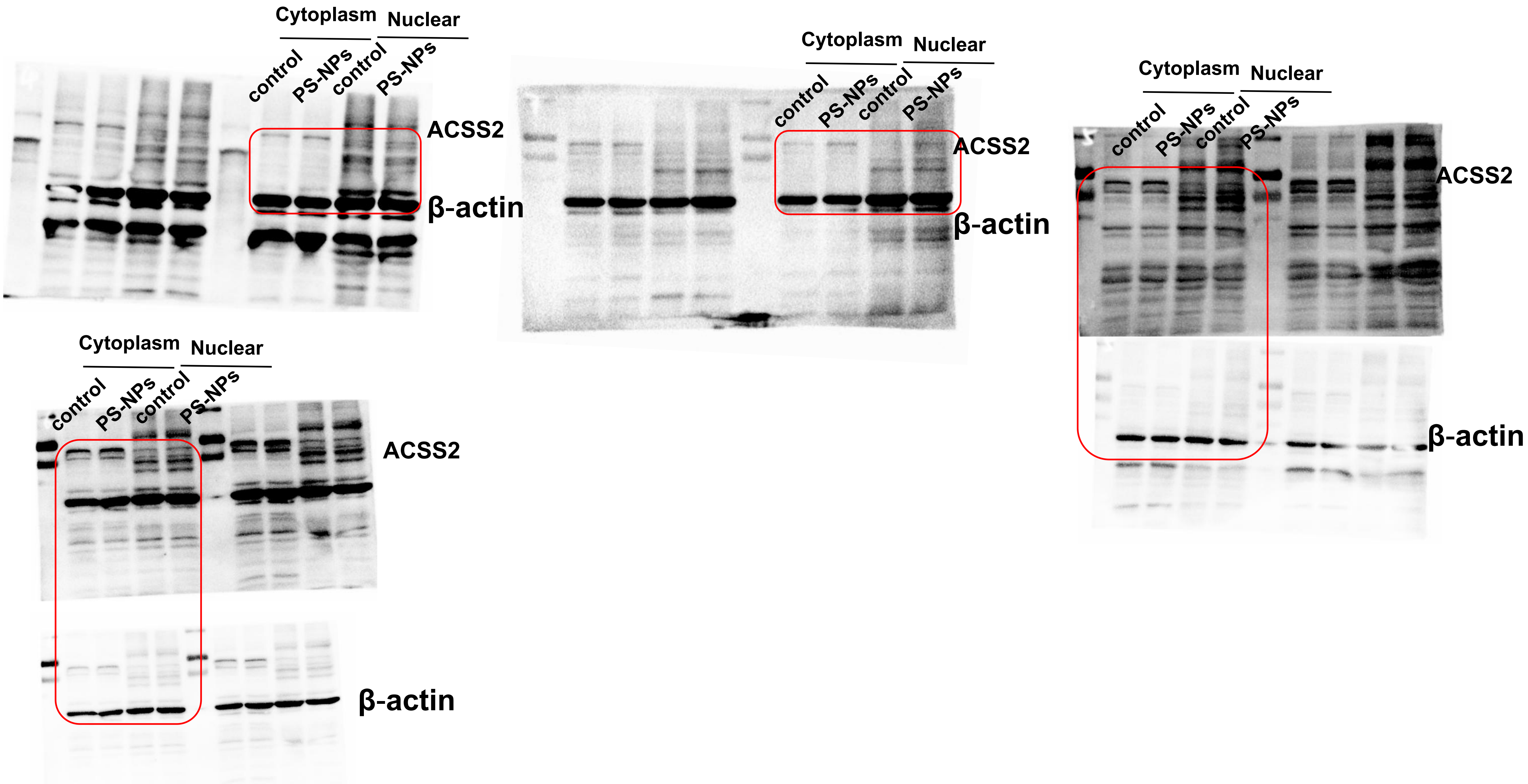

Nucleoplasmic separation HEC-1B control vs. HEC-1B compoundC(ACSS2)

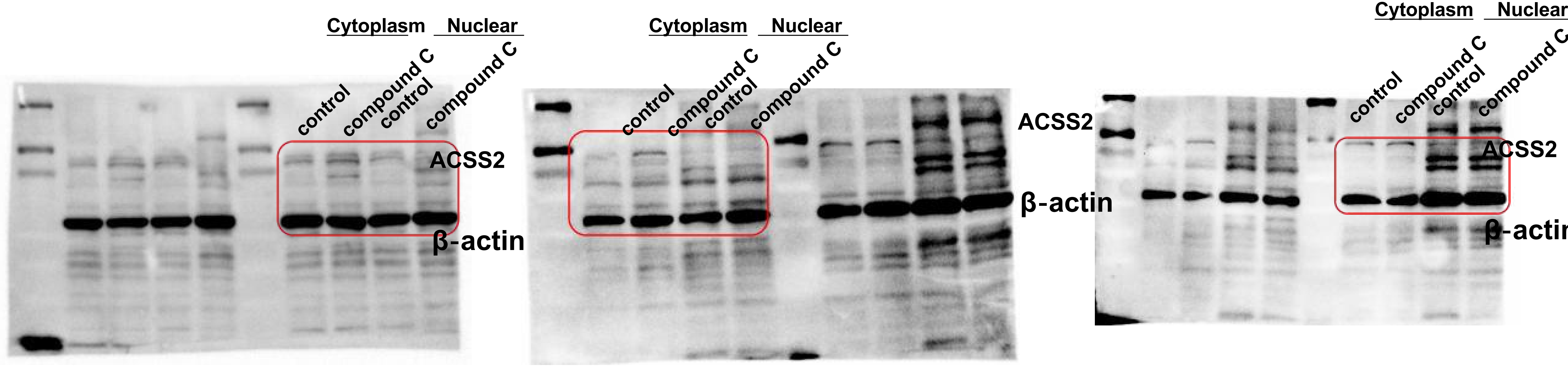

HEC-1B control vs. HEC-1B PS-NPs treated (H3K9ac)

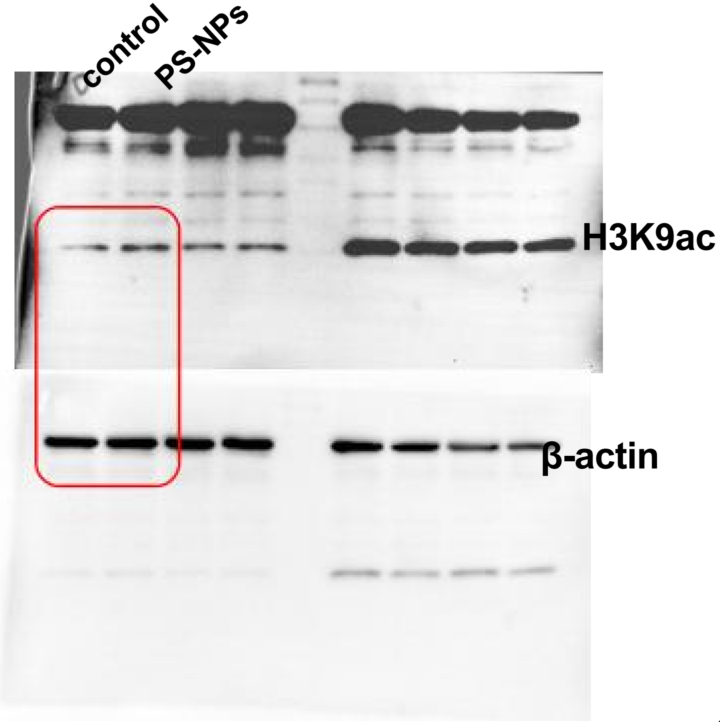

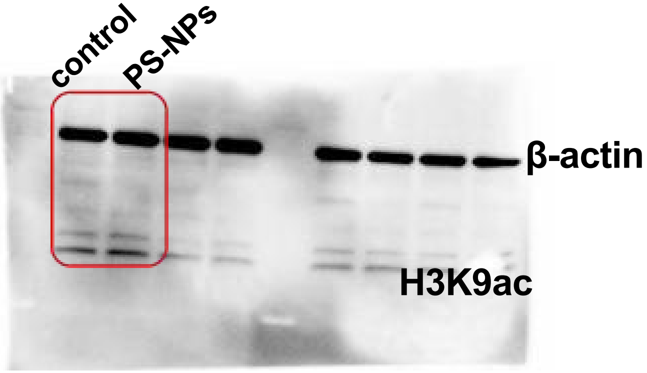

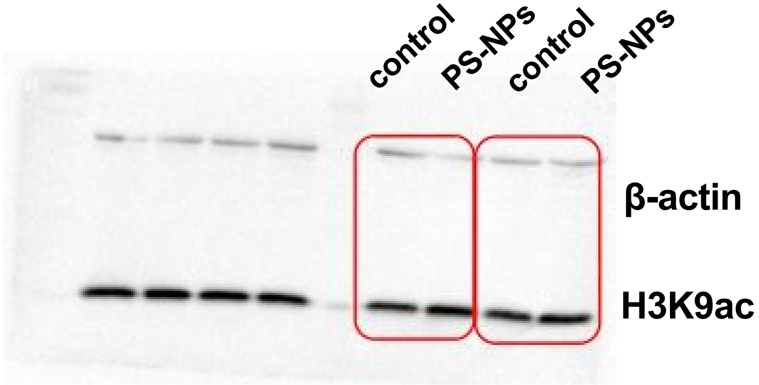

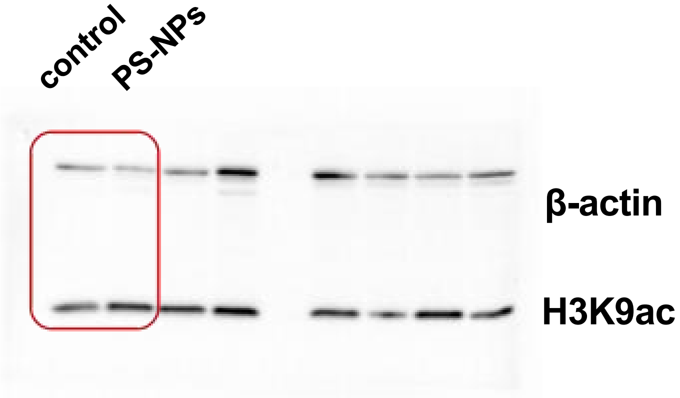

HEC-1B PS-NPs si-NC vs. HEC-1B PS-NPs si-ACSS2 (H3K9ac)

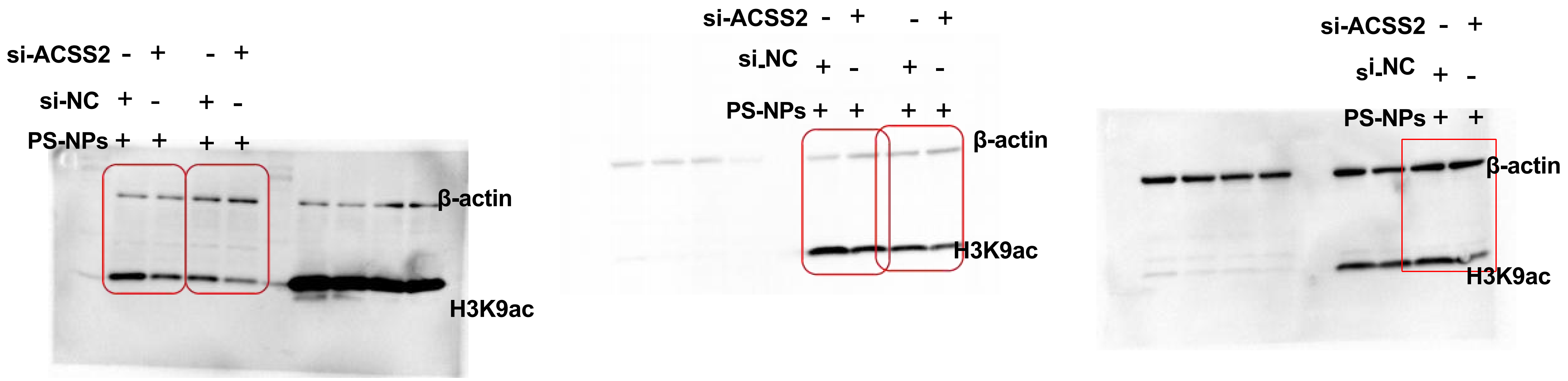

HEC-1B control vs. HEC-1B PS-NPs treated (PLA2G3)

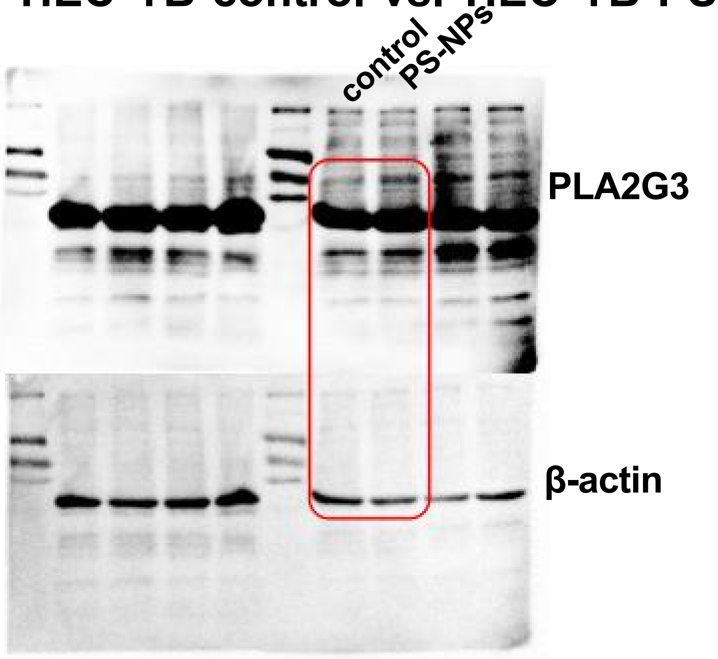

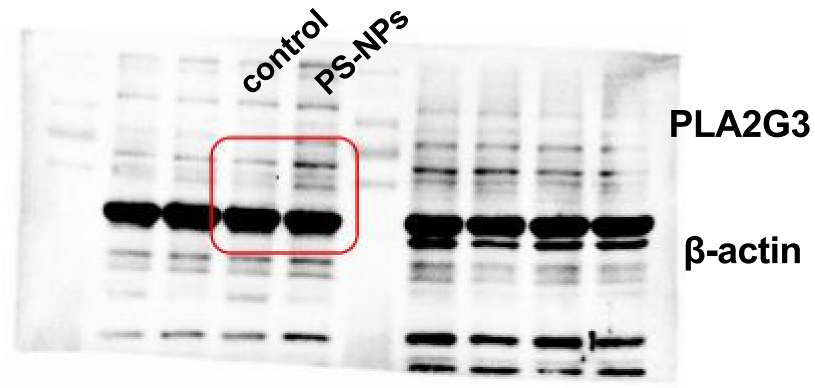

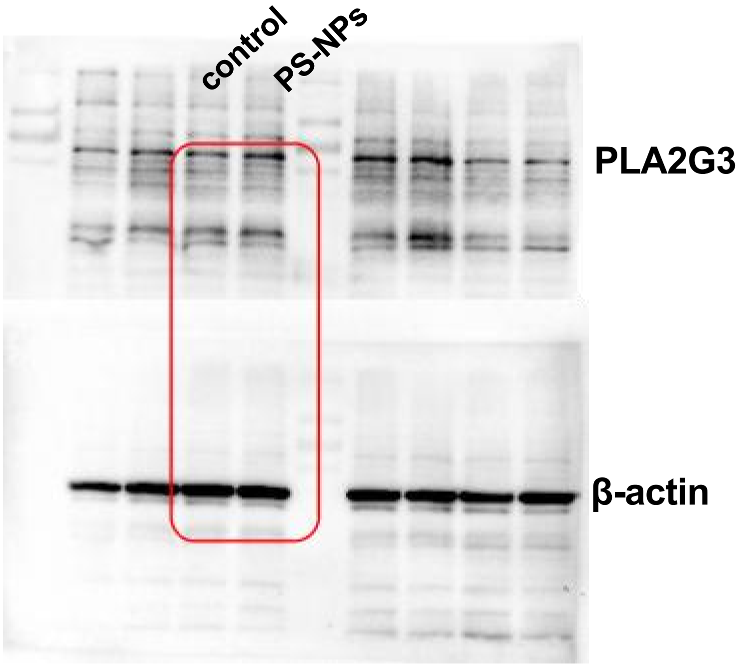

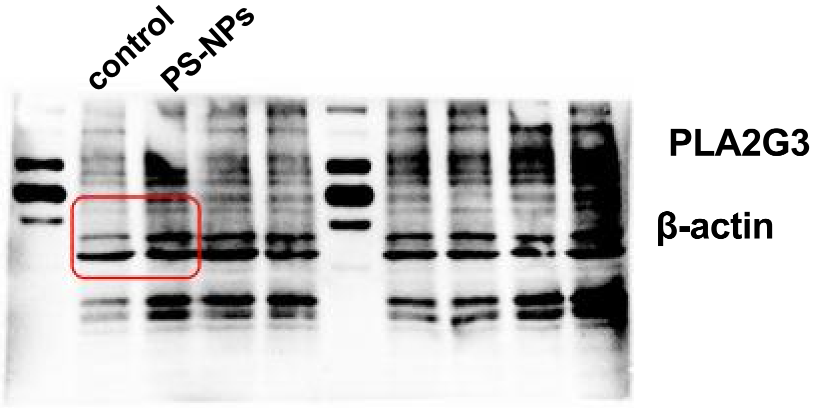

HEC-1B PS-NPs si-NC vs. HEC-1B PS-NPs si-ACSS2 (PLA2G3)

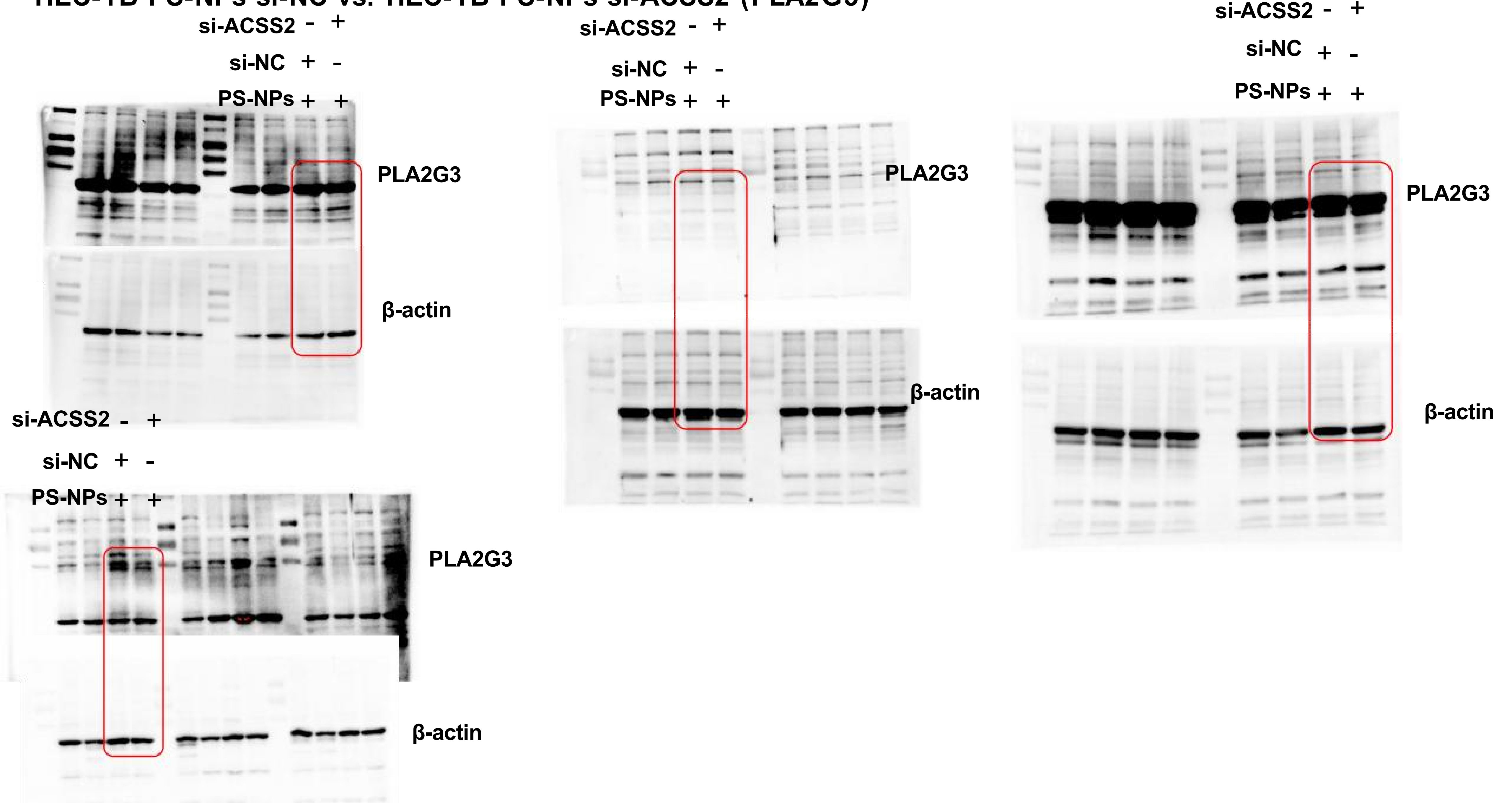

HEC-1B control vs. HEC-1B PS-NPs treated (E-cadherin)

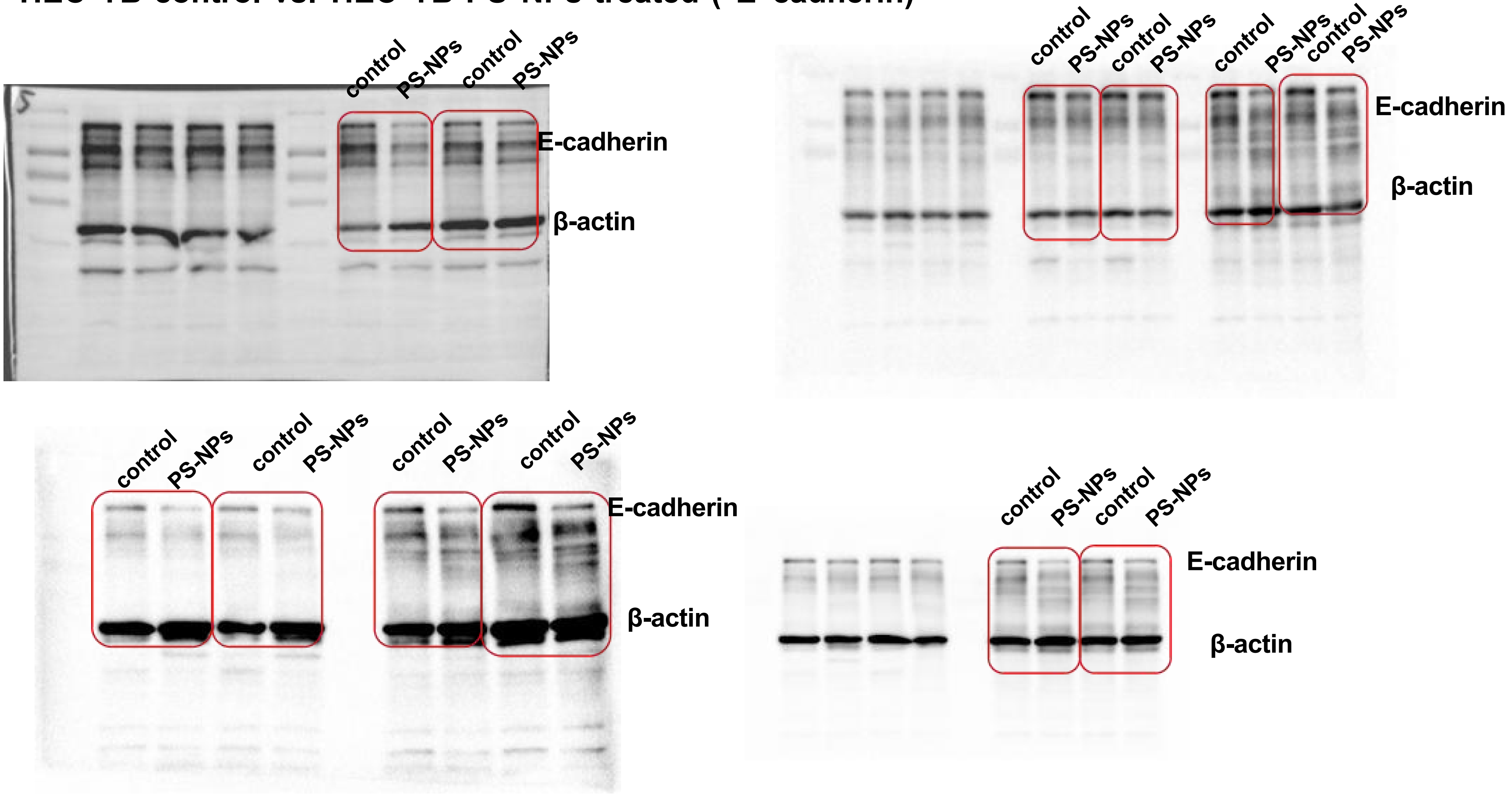

HEC-1B control+si-NC vs. HEC-1B PS-NPs treated si-NC vs. HEC-1B PS-NPs treated si-PLA2G3 (E-cadherin)

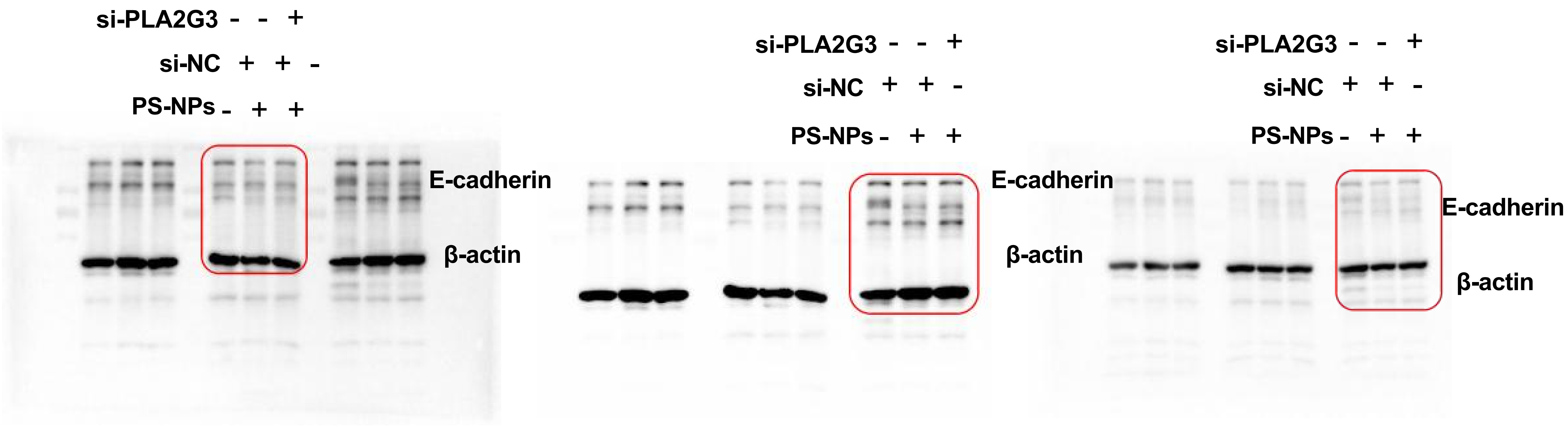

HEC-1B control vs. HEC-1B PS-NPs treated (N-cadherin)

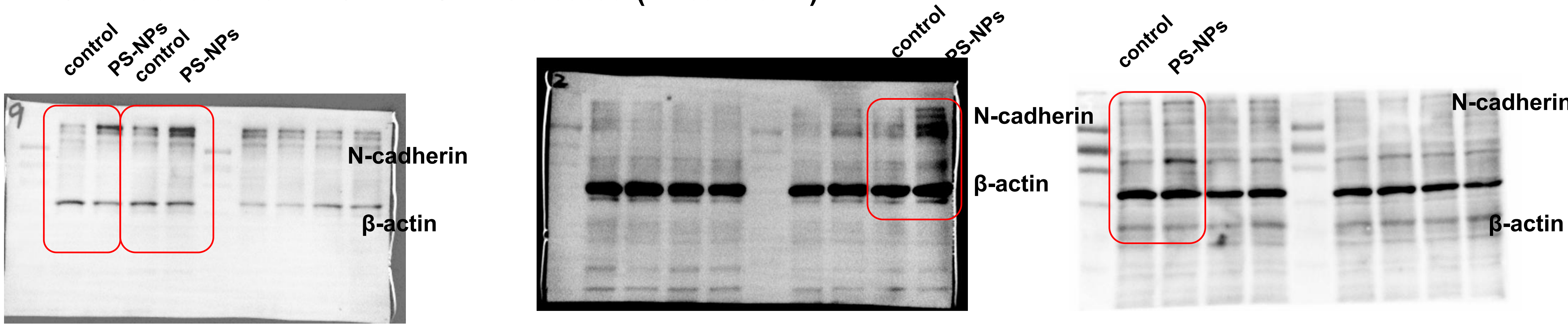

HEC-1B control+si-NC vs. HEC-1B PS-NPs treated si-NC vs. HEC-1B PS-NPs treated si-PLA2G3 (N-cadherin)

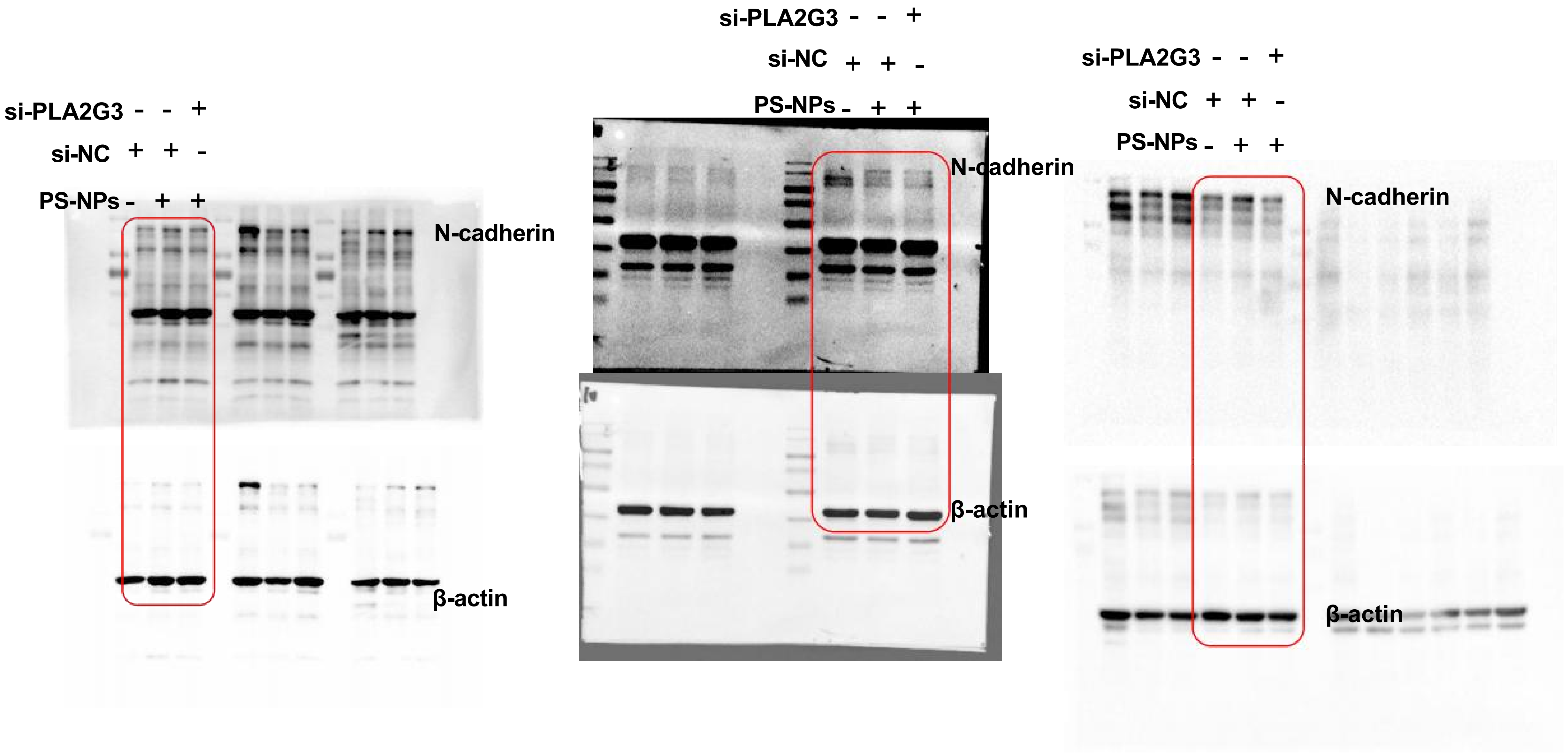

HEC-1B control vs. HEC-1B PS-NPs treated vs. HEC-1B PS-NPs treated+4-PBA (AMPKα pT172)

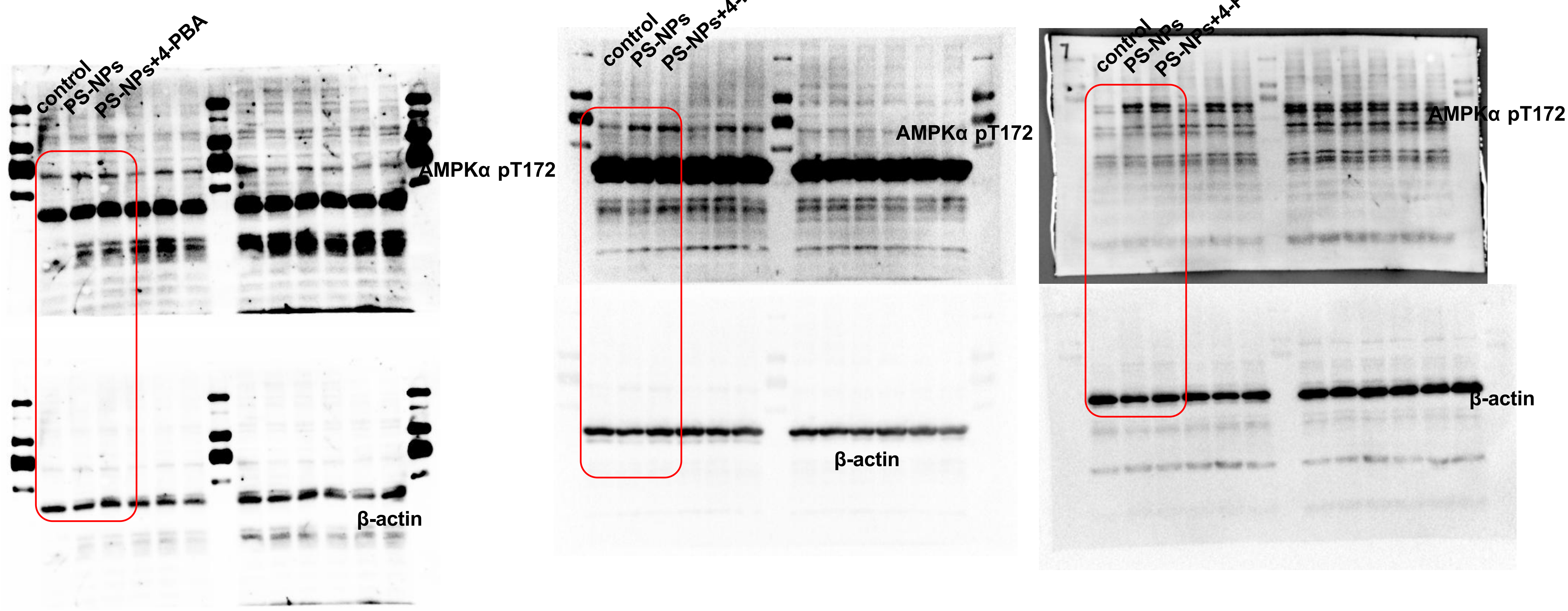

HEC-1B control vs. HEC-1B PS-NPs treated vs. HEC-1B PS-NPs treated+NAC(AMPKα pT172)

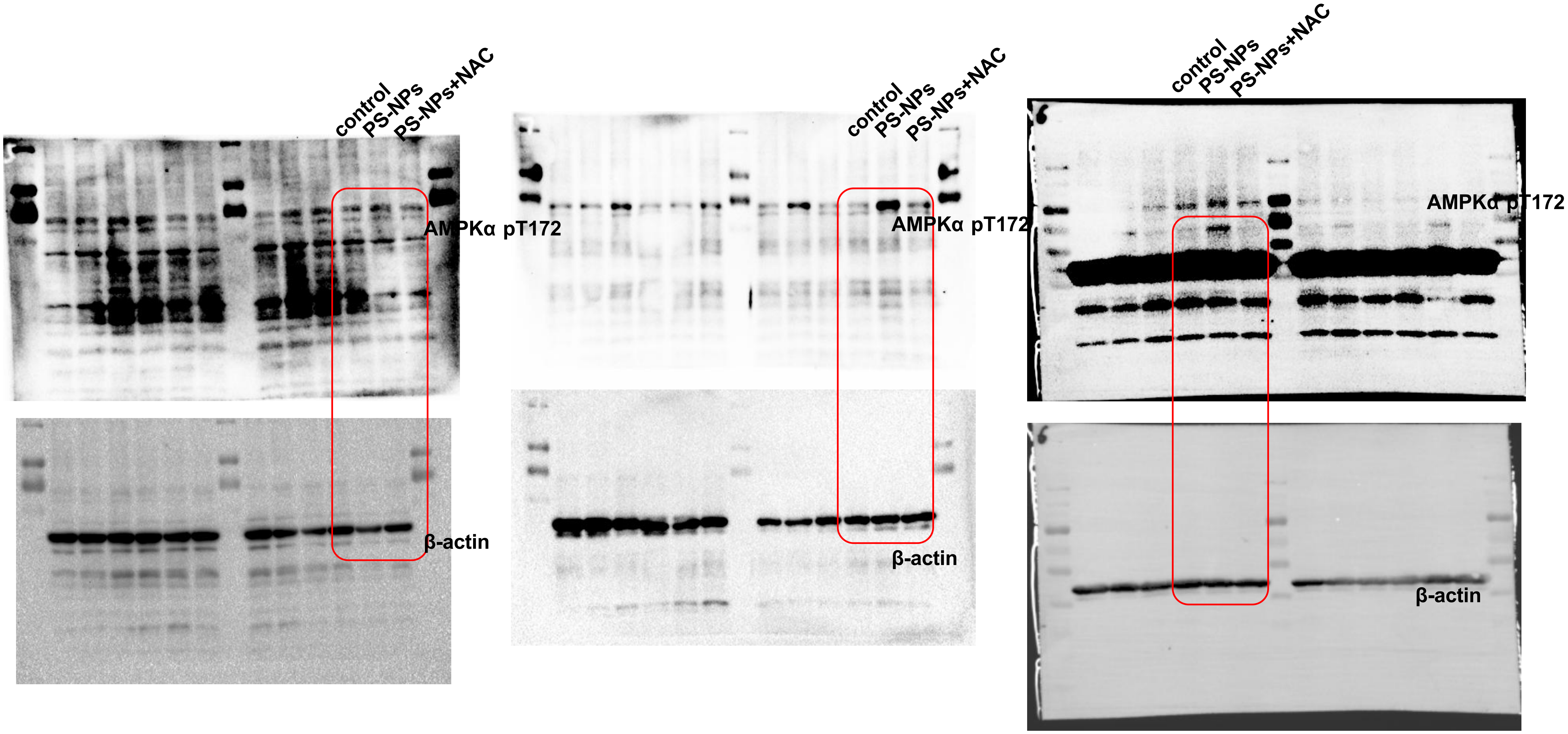

HEC-1B control vs. HEC-1B PS-NPs treated (β-catenin)

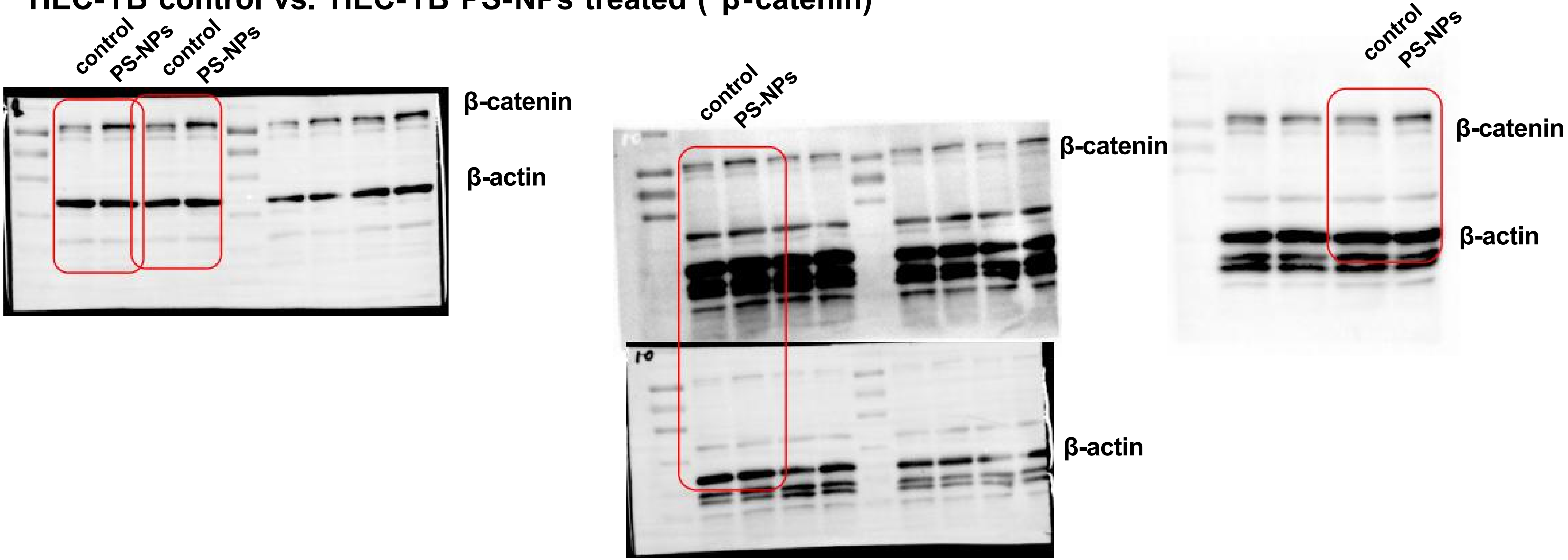

HEC-1B control+si-NC vs. HEC-1B PS-NPs treated si-NC vs. HEC-1B PS-NPs treated si-PLA2G3 (β-catenin)

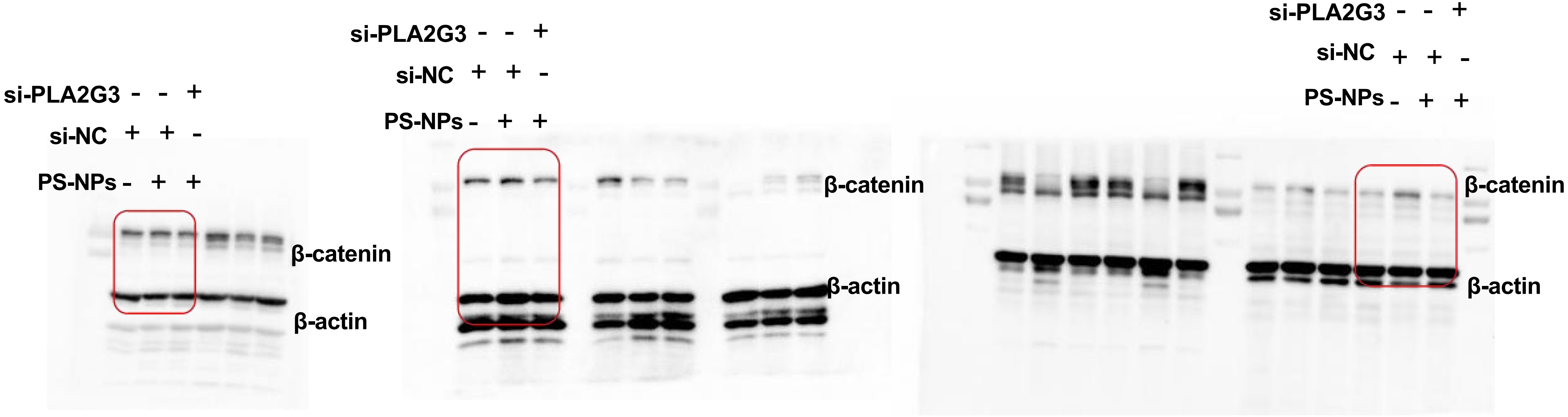

Mouse protein expression: HEC-1B control vs. HEC-1B PS-NPs treated (ACSS2,PLA2G3)

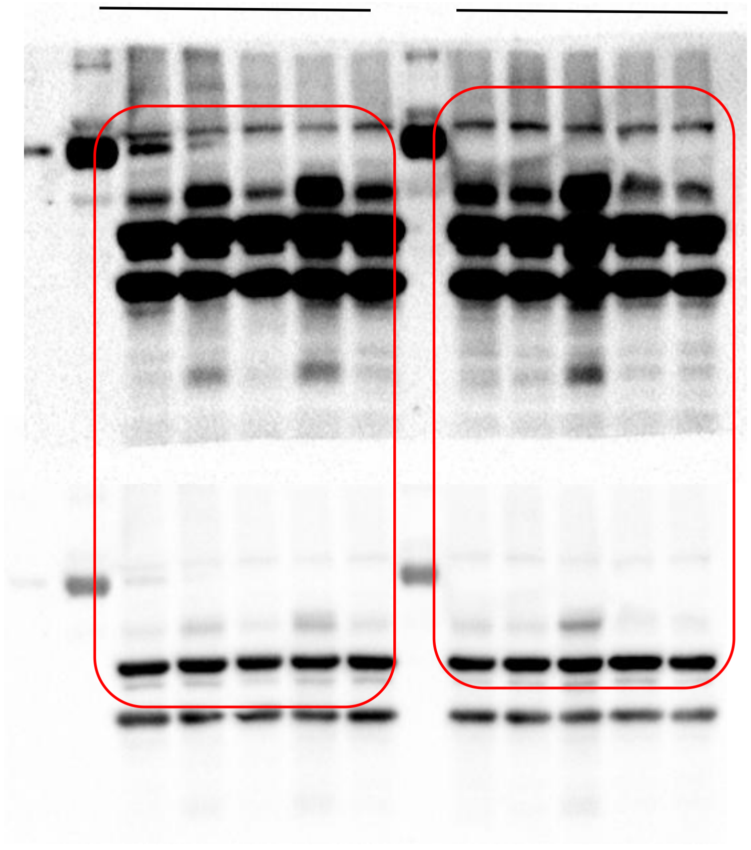

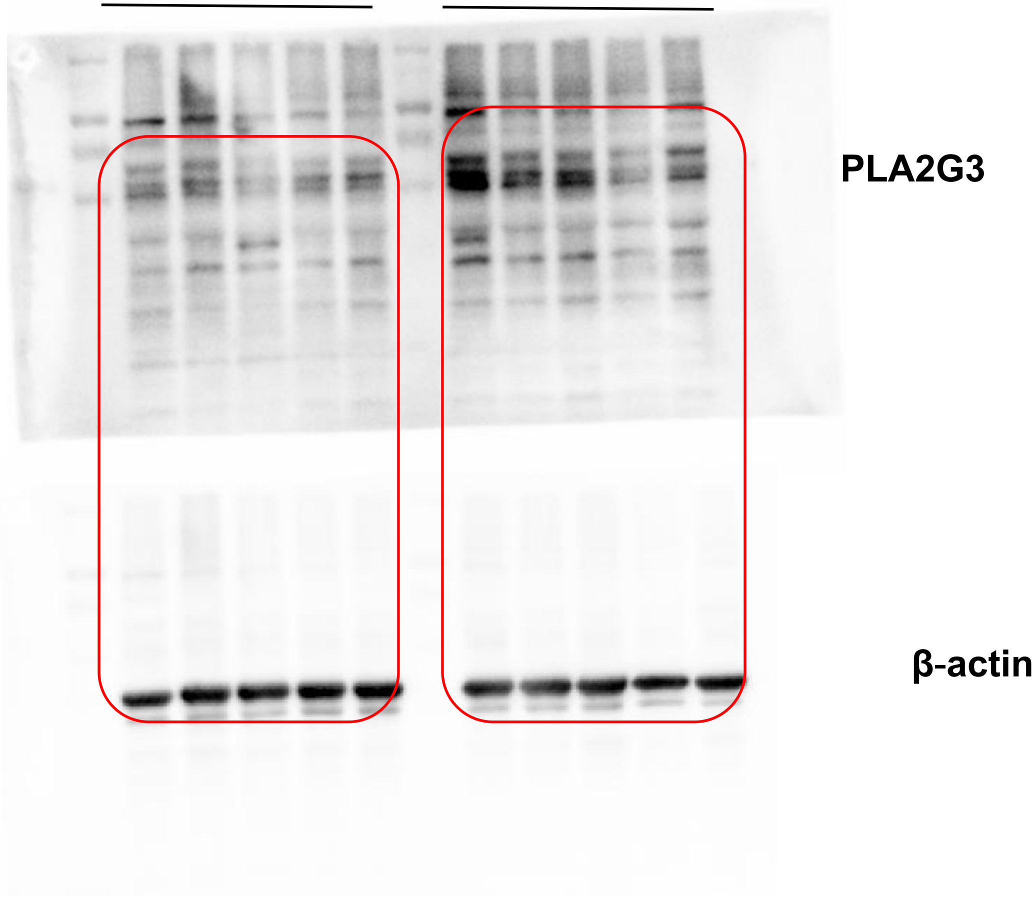

Mouse protein expression: HEC-1B control vs. HEC-1B PS-NPs treated (E-cadherin，N-cadherin)

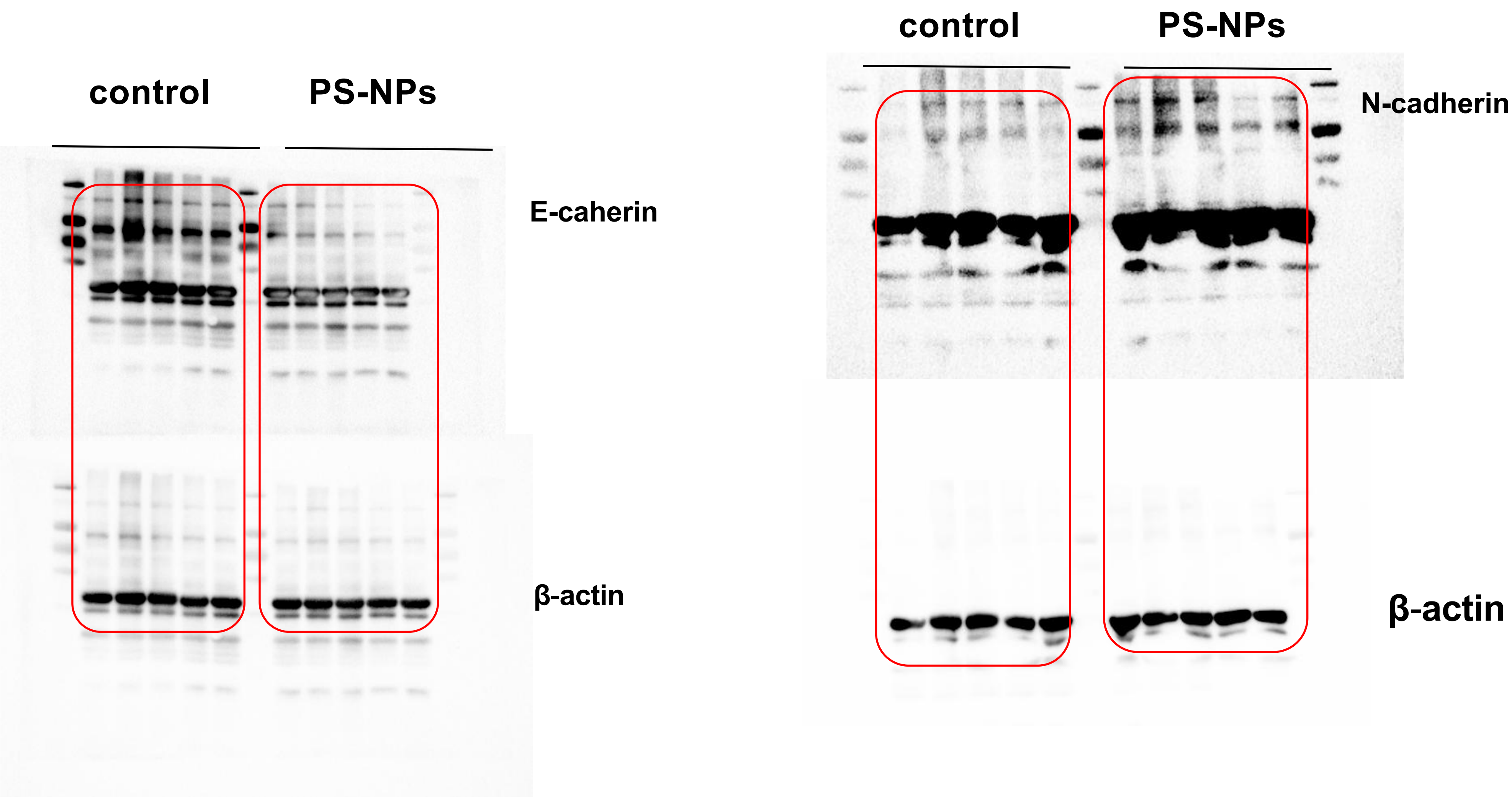

Mouse protein expression: HEC-1B control vs. HEC-1B PS-NPs treated (ER, PR)

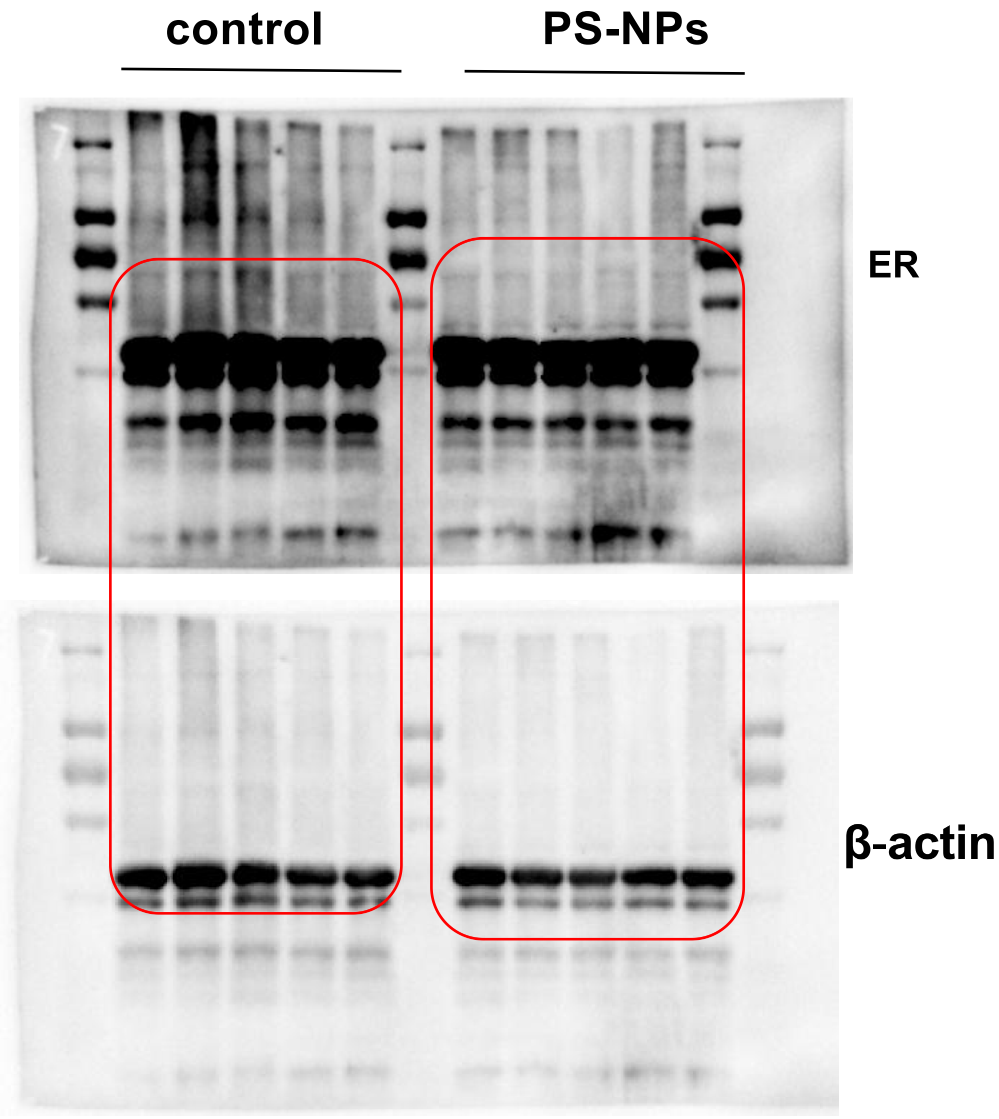

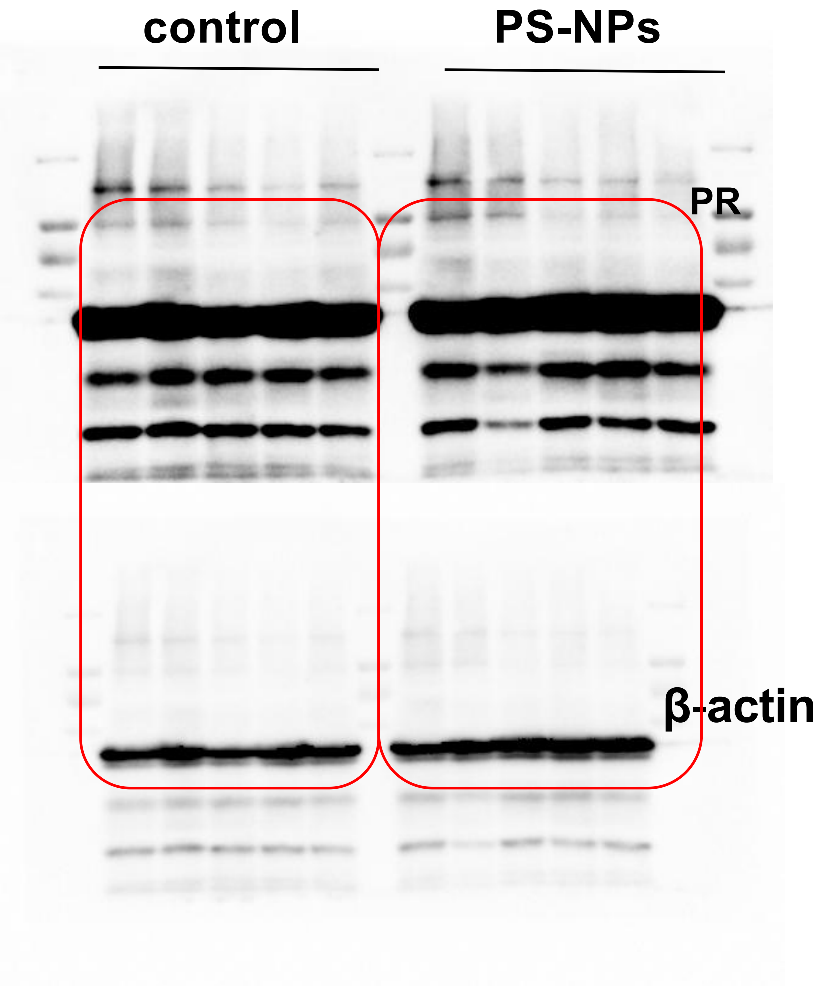

Mouse protein expression: HEC-1B control vs. HEC-1B PS-NPs treated (MMP2, α-SMA)

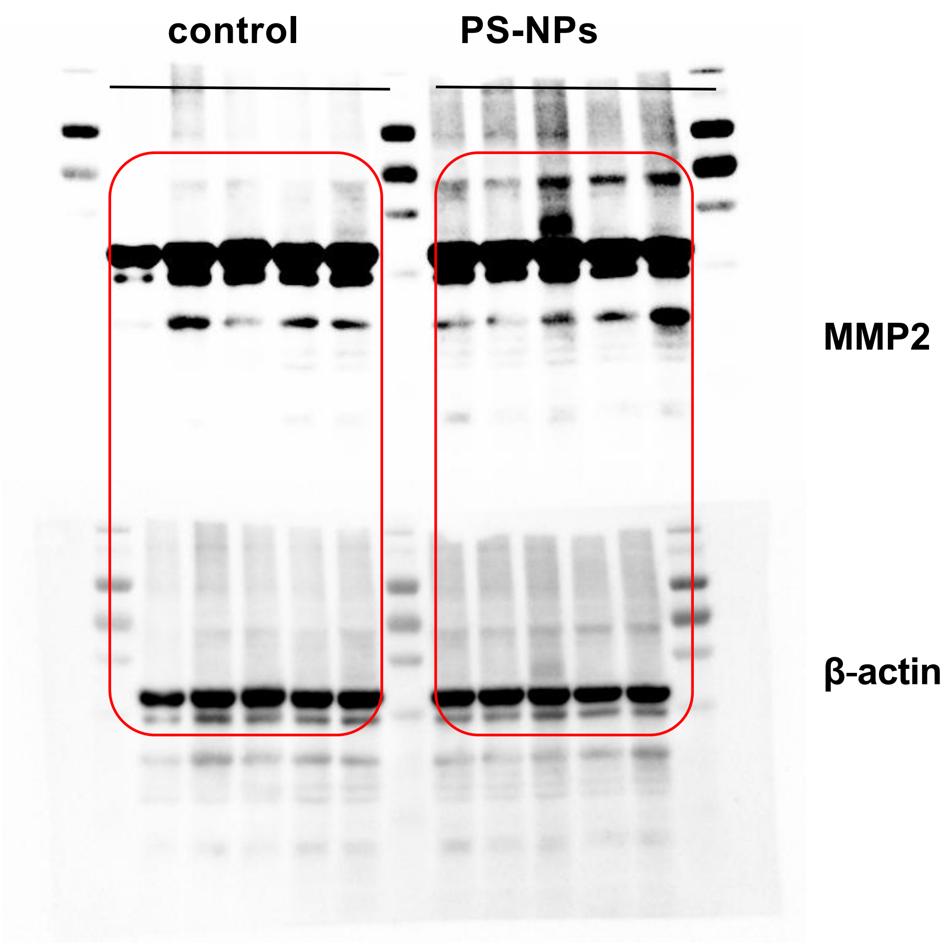

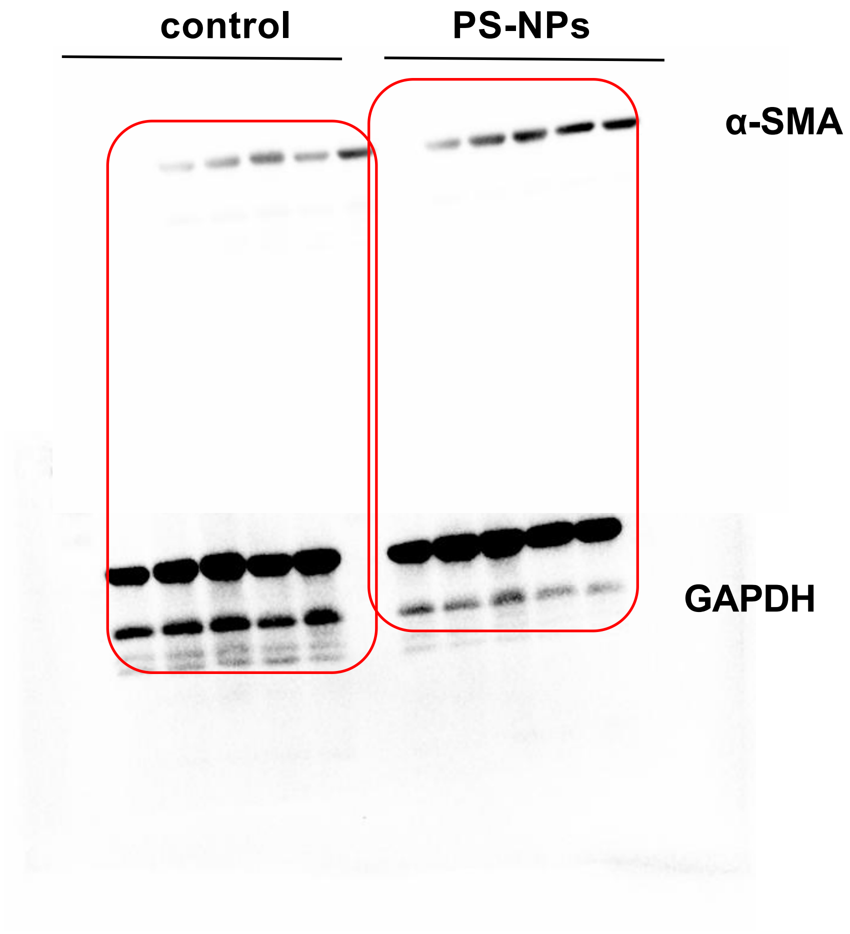

Supplement: Supplementary file 2 — original Western blot [file 41420_2026_3071_MOESM2_ESM.pdf]
